# Low-intensity cognitive-behaviour therapy interventions for obsessive-compulsive disorder compared to waiting list for therapist-led cognitive-behaviour therapy: 3-arm randomised controlled trial of clinical effectiveness

**DOI:** 10.1371/journal.pmed.1002337

**Published:** 2017-06-27

**Authors:** Karina Lovell, Peter Bower, Judith Gellatly, Sarah Byford, Penny Bee, Dean McMillan, Catherine Arundel, Simon Gilbody, Lina Gega, Gillian Hardy, Shirley Reynolds, Michael Barkham, Patricia Mottram, Nicola Lidbetter, Rebecca Pedley, Jo Molle, Emily Peckham, Jasmin Knopp-Hoffer, Owen Price, Janice Connell, Margaret Heslin, Christopher Foley, Faye Plummer, Christopher Roberts

**Affiliations:** 1 Division of Nursing, Midwifery and Social Work, School of Health Sciences, Faculty of Biology, Medicine and Health, The University of Manchester, Manchester Academic Health Science Centre, Manchester, United Kingdom; 2 NIHR School for Primary Care Research, University of Manchester, Manchester, United Kingdom; 3 King's Health Economics, Institute of Psychiatry, Psychology & Neuroscience, King's College London, London, United Kingdom; 4 Hull York Medical School & Department of Health Sciences, University of York, York, United Kingdom; 5 Department of Health Sciences, University of York, York, United Kingdom; 6 Centre for Psychological Services Research, Department of Psychology, University of Sheffield, Sheffield, United Kingdom; 7 School of Psychology, University of Reading, Reading, United Kingdom; 8 Cheshire & Wirral Partnership, NHS Foundation Trust, Wallasey, United Kingdom; 9 Anxiety UK, Manchester, United Kingdom; 10 Norwich Medical School, University of East Anglia, Norwich, United Kingdom; 11 ScHARR, University of Sheffield, Sheffield, United Kingdom; 12 Department of Public Health & Primary Care, Cambridge University, Cambridge, United Kingdom; 13 Bradford Institute for Health Research, Bradford, United Kingdom; 14 Centre for Biostatistics, University of Manchester, Manchester, United Kingdom; Western Sydney University, AUSTRALIA

## Abstract

**Background:**

Obsessive-compulsive disorder (OCD) is prevalent and without adequate treatment usually follows a chronic course. “High-intensity” cognitive-behaviour therapy (CBT) from a specialist therapist is current “best practice.” However, access is difficult because of limited numbers of therapists and because of the disabling effects of OCD symptoms. There is a potential role for “low-intensity” interventions as part of a stepped care model. Low-intensity interventions (written or web-based materials with limited therapist support) can be provided remotely, which has the potential to increase access. However, current evidence concerning low-intensity interventions is insufficient. We aimed to determine the clinical effectiveness of 2 forms of low-intensity CBT prior to high-intensity CBT, in adults meeting the Diagnostic and Statistical Manual of Mental Disorders, Fourth Edition (DSM-IV) criteria for OCD.

**Methods and findings:**

This study was approved by the National Research Ethics Service Committee North West–Lancaster (reference number 11/NW/0276). All participants provided informed consent to take part in the trial. We conducted a 3-arm, multicentre randomised controlled trial in primary- and secondary-care United Kingdom mental health services. All patients were on a waiting list for therapist-led CBT (treatment as usual). Four hundred and seventy-three eligible patients were recruited and randomised. Patients had a median age of 33 years, and 60% were female. The majority were experiencing severe OCD. Patients received 1 of 2 low-intensity interventions: computerised CBT (cCBT; web-based CBT materials and limited telephone support) through “OCFighter” or guided self-help (written CBT materials with limited telephone or face-to-face support). Primary comparisons concerned OCD symptoms, measured using the Yale-Brown Obsessive Compulsive Scale–Observer-Rated (Y-BOCS-OR) at 3, 6, and 12 months. Secondary outcomes included health-related quality of life, depression, anxiety, and functioning. At 3 months, guided self-help demonstrated modest benefits over the waiting list in reducing OCD symptoms (adjusted mean difference = −1.91, 95% CI −3.27 to −0.55). These effects did not reach a prespecified level of “clinically significant benefit.” cCBT did not demonstrate significant benefit (adjusted mean difference = −0.71, 95% CI −2.12 to 0.70). At 12 months, neither guided self-help nor cCBT led to differences in OCD symptoms. Early access to low-intensity interventions led to significant reductions in uptake of high-intensity CBT over 12 months; 86% of the patients allocated to the waiting list for high-intensity CBT started treatment by the end of the trial, compared to 62% in supported cCBT and 57% in guided self-help. These reductions did not compromise longer-term patient outcomes. Data suggested small differences in satisfaction at 3 months, with patients more satisfied with guided self-help than supported cCBT. A significant issue in the interpretation of the results concerns the level of access to high-intensity CBT before the primary outcome assessment.

**Conclusions:**

We have demonstrated that providing low-intensity interventions does not lead to clinically significant benefits but may reduce uptake of therapist-led CBT.

**Trial registration:**

International Standard Randomized Controlled Trial Number (ISRCTN) Registry ISRCTN73535163.

## Abstract 2

Karina Lovell and colleagues reveal that low-intensity computerised cognitive behaviour therapy provides no clinical benefit to OCD patients.

## Author summary

**Why was this study done?:**

- Many people suffer from obsessive-compulsive disorder (OCD), and if they do not get treatment, it can be a long-term problem.
- Although cognitive-behaviour therapy (CBT) with a therapist is effective, many people struggle to get access because of limited numbers of therapists.
- Low-intensity versions of CBT (written or web-based materials with limited therapist support) may increase access to care, but evidence of their effectiveness is limited.

**What did the researchers do and find?:**

- We tested 2 low-intensity versions of CBT in a trial (guided self-help and supported computerised cognitive-behaviour therapy [cCBT]), testing their impact on patients with OCD when provided prior to CBT with a therapist.
- Neither low-intensity version of CBT led to clinically significant benefits in patient outcomes.
- Access to “low-intensity” interventions led to significant reductions in uptake of CBT with a therapist over 12 months.
- More patients were satisfied with guided self-help than supported cCBT.

**What do these findings mean?:**

- Providing low-intensity interventions does not lead to clinically significant benefits but may reduce uptake of therapist-led CBT.
- These findings from a large pragmatic trial showing no clinical benefit from low-intensity treatments are in contrast to other studies published recently.

## Introduction

Obsessive-compulsive disorder (OCD) has an estimated lifetime prevalence of 2%–3% and is rated among the top 10 causes of disability worldwide, with an estimated US$8.4 billion attributable to OCD in the United States [1]. Providing accessible and effective care for OCD is a priority worldwide.

However, there is evidence that people with OCD struggle to access treatment, with consistent reports of a marked delay between OCD onset and management. One study found a 10-year gap between onset and seeking help and 17 years between onset and receiving effective help [2].

In OCD, both medication and psychological therapy are effective, with the “gold standard” psychological therapy intervention being therapist-led cognitive-behaviour therapy (CBT) [3], with 1-hour weekly sessions delivered predominantly face-to-face over 12–16 weeks. However, it is relatively costly, and the limited availability of specialist therapists means that access can be poor, with long waiting times. Additionally, OCD is characterised by intrusive, unwanted, recurrent, and distressing thoughts, images, or impulses (i.e., obsessions) and repetitive actions or rituals (compulsions). These obsessions and compulsions can make it more difficult for patients to engage with treatment because of fears of contamination or causing harm to others.

Conventional ways of delivering psychological therapy are being challenged. Health systems under financial pressure need to manage demand more effectively through new methods of delivery [4], and innovation is needed to meet the needs of diverse patient populations with complex needs. Research has shown how conventional therapist-led CBT can be delivered effectively in low-intensity forms including guided self-help (written CBT materials with limited telephone or face-to-face support) or computerised CBT (cCBT; web-based CBT materials and limited telephone support). Both forms are potentially cheaper and more accessible than conventional therapist-led CBT and demonstrate evidence of effectiveness in a range of disorders [5,6]. Low-intensity CBT interventions for OCD may provide more rapid relief of symptoms, reduce the need for more expensive therapist-led CBT, and encourage more efficient use of healthcare resources when delivered as part of a stepped care system [7].

At the time this study was commissioned, the evidence base for low-intensity interventions in OCD was far from definitive. Much of the evidence for guided self-help was based on small open or uncontrolled studies [8,9] or comparisons of nonguided self-help with guided self-help [10]. A systematic review of cCBT for OCD found only 4 studies [11]. cCBT reduced rituals and obsessions and improved functioning and was more effective than attention control [12], but not as effective as therapist-led CBT. Clearly, the potential benefits of both guided self-help and cCBT need to be demonstrated in large-scale trials.

We conducted a randomised trial for patients with OCD, allocating patients to either (a) guided self-help prior to therapist-led CBT, (b) cCBT prior to therapist-led CBT, or (c) a waiting list for therapist-led CBT only. We aimed to provide a definitive answer to the following questions:

1. Does access to guided self-help or cCBT provide more rapid improvement in OCD symptoms at 3 months compared to a waiting list for therapist-led CBT?
2. Does access to guided self-help or cCBT improve patient satisfaction at 3 months compared to a waiting list for therapist-led CBT?
3. Does access to guided self-help or cCBT prior to therapist-led CBT provide longer-term improvement in OCD symptoms at 12 months compared to therapist-led CBT alone?
4. Does access to guided self-help or cCBT reduce uptake of therapist-led CBT over 12 months?

## Methods

This study was approved by the National Research Ethics Service Committee North West–Lancaster (reference number 11/NW/0276). All participants provided informed consent to take part in the trial.

### Study design and participants

We conducted a pragmatic trial, delivered in routine service settings, to provide a balance between internal and external validity and maximise relevance to clinical guidelines [13,14]. The study protocol has been published [15]. The study is reported as per Consolidated Standards of Reporting Trials (CONSORT) guidelines, as described in the CONSORT checklist (S2 Text). Potential participants were most frequently identified by administrative and clinical staff in primary-and secondary-care screening waiting lists, although self-referral was used at 1 site to increase recruitment (via adverts in newspapers, community facilities, and social media). Potential participants were provided with an information pack. Those who provided consent to contact took part in a telephone eligibility screen to determine if they were over 18 years old and not currently receiving a psychological therapy for OCD or experiencing severe and distressing psychotic symptoms. Participants passing the initial screen were offered a face-to-face eligibility appointment.

We included patients who were (1) aged 18+ years, (2) able to read English, (3) currently waiting for access to therapist-led CBT, (4) meeting DSM-IV criteria for OCD (assessed using the Mini-International Neuropsychiatric Interview [16]), and (5) scoring 16+ on the Yale-Brown Obsessive Compulsive Checklist–Self-Report (Y-BOCS-SR [17]).

We excluded patients (1) experiencing active suicidal or psychotic thoughts, (2) meeting DSM-IV alcohol or substance dependence criteria, (3) receiving psychological treatment for OCD, or (4) with language difficulties that would preclude participation.

### Randomisation and masking

Patients were randomised (ratio 1:1:1) via a secure web system independently administered by a trials unit to ensure concealment of allocation. Allocation was minimised on OCD severity, OCD duration, antidepressant use, and depression severity. It was not possible to mask participants or clinical staff to treatment allocation. Research staff undertaking assessments were masked to allocation: unmasking was reported in 30%, 22%, and 26% of the 3, 6, and 12 month interviews, respectively. When this occurred, subsequent assessments were done by another researcher to limit bias.

### Procedures

cCBT *w*as delivered using OCFighter (www.ccbt.co.uk), a commercial OCD program. OCFighter involves 9 steps (focussed on exposure and response prevention) to help people with OCD carry out treatment and monitor progress. Participants received a secure login and were advised to use cCBT at least 6 times over 12 weeks. Participants received six 10-minute telephone calls, for risk assessment, progress review, and problem solving.

Guided self-help was delivered using the book *Obsessive Compulsive Disorder*: *A Self-Help Book* [18], which is focussed on exposure and response prevention. Participants received weekly guidance, with an initial session (60 minutes face to face or by telephone, dependent on patient preference) followed by 10 30-minute sessions over 12 weeks. The support involved an explanation of the workbook, help devising goals, risk assessment, support for conducting CBT homework, progress review, and problem solving.

Support for both cCBT and guided self-help was provided by “psychological well-being practitioners.” These are graduates with no prior clinical qualifications who receive 12 months training and who are responsible for delivering guided self-help CBT and general education for anxiety and depression in England. Most have limited OCD-specific training. They were trained in both interventions over 3 days by the research team (with additional support from the company supplying cCBT). All staff received telephone supervision every fortnight from the research team or from experienced therapists within routine services. In total, 93 psychological well-being practitioners managed patients in the trial (range 1–18 patients), with 46 practitioners allocated patients in both interventions. Psychological well-practitioner characteristics are reported in Table A in S1 Appendix.

Psychological well-being practitioners recorded dates, length, and mode of contact for all sessions and were asked to record sessions to examine fidelity. We also received automated recordings of cCBT use. Fidelity was evaluated by an independent rater blind to outcome. We defined tasks to be carried out in both interventions, which were coded from recordings as “implicit,” “explicit,” or “absent,” and an overall rating was generated using a 5-point scale (“unacceptable” to “excellent”).

The comparator was waiting list for therapist-led CBT. As the trial was a pragmatic design within routine services, we were unable to mandate a waiting period for therapist-led CBT. We expected that most patients would start therapist-led CBT 3–6 months following allocation, after receiving their low-intensity interventions (where allocated). Therapist-led CBT was typically 8–20 face-to-face, 45–60-minute weekly sessions.

In this pragmatic trial, we placed no restrictions on treatment after randomisation. Before seeing a therapist, patients on waiting lists sometimes received interventions other than our low-intensity interventions, including education, medication, or nonspecific interventions (such as “stress management”). All additional care outside the trial protocol was recorded as part of the economic evaluation.

### Clinical outcomes

We conducted follow-up assessments at 3 months (primary outcome timepoint), 6 months, and 12 months following randomisation. The primary outcome measure, Yale-Brown Obsessive Compulsive Scale–Observer Rated (Y-BOCS-OR) [17], was collected face to face at baseline. At follow-up time points where face-to-face collection was not possible following a highly structured standardised operating procedure, telephone or postal assessment using the Y-BOCS-SR was attempted.

The Y-BOCS-OR is an interview-administered structured assessment that provides an indication of OCD symptom severity. It consists of 2 comprehensive symptom checklists exploring current and past symptoms of obsession and compulsion (over the past week and past symptoms) and a 10-item severity scale exploring current obsessive and compulsive symptoms. Impairment over 5 clinical domains is identified: time consumed, functional impairment, psychological distress, efforts to resist, and perceived sense of control on a 5-point Likert scale from 0 (none) to 4 (extreme). Scores from the 10 items are summed to identify level of severity (0–7 subclinical, 8–15 mild, 16–23 moderate, 24–31 severe, and 32–40 extreme).

Secondary outcomes were collected at baseline and at the 3, 6, and 12-month follow-up. Outcomes included the Y-BOCS-SR, a self-report version of the Y-BOCS-OR scale. When it was not possible to complete the Y-BOCS-OR, the Y-BOCS-SR was used as a proxy.

Other secondary outcomes (all self-report) included the Short Form-36 (SF-36) [19] for health-related quality of life; Clinical Outcomes in Routine Evaluation (CORE-OM) [20] for distress; Patient Health Questionnaire (PHQ-9) [21] for depression; Generalized Anxiety Disorder 7-item (GAD-7) scale [22] for generalised anxiety disorder; Work and Social Adjustment Scale (WSAS) [23] for functional impairment; IAPT Employment Status Questions (A13-A14) [24] for employment rates and receipt of statutory sick pay; and the Client Satisfaction Questionnaire (CSQ-8-UK) [25] for satisfaction. Comorbidities (Clinical Interview Schedule-Revised; CIS-R) [26] and demographics were collected at baseline only.

### Statistical analysis

With 3 pair-wise comparisons, alpha was set at 1.67%. We assumed a standard deviation for the primary outcome of 7.3, a correlation between baseline and follow up of 0.43 [27], and a therapist intracluster correlation (ICC) between therapists (0.06) and within therapist (0.015). Assuming 85% retention, 432 patients were required. Trial monitoring suggested a lower follow-up rate, and thus, the sample size was increased to 473 to retain power. In total, 366 patients at follow-up provided power greater than 80% to detect a clinically significant difference of 3 Y-BOCS points for each comparison.

Preliminary modelling determined methods for handling missing data (full details provided in the statistical analysis plan: https://dx.doi.org/10.6084/m9.figshare.3503885). There was no deviation from the prespecified plan, all analyses were conducted, and those not present in this manuscript are reported in the Health Technology Assessment (HTA) report [28]. Missing baseline covariates were imputed by single imputation [29] using other covariates. Analyses of the primary outcome were based on a linear mixed model with random effects for psychological well-being practitioners. As practitioners were crossed with treatment, correlated random effects were included for each treatment, enabling estimation of the ICC for cCBT and guided self-help. We included the following covariates: OCD duration and severity; anxiety, depression, antidepressant use; and gender. Binary outcomes (e.g., uptake of therapist-led CBT) used logistic regression to estimate adjusted odds ratios (ORs), with the same baseline covariates. Analysis used intention-to-treat subject to the availability of data. Distributional assumptions of the models were checked. All outcomes included in the Obsessive Compulsive Treatment Efficacy Trial (OCTET) protocol are detailed.

### Patient involvement

Patients and members of the public were involved throughout the trial, including the design, management, and conduct of the trial. From the outset, the chief executive of a national user-led organisation (Anxiety UK) was involved as a coapplicant and collaborator. Members of an OCD self-help group assisted with the development of the guided self-help manual and adaptations to one of the trial outcome measures. A service user with OCD was a member of the trial steering committee, while another conducted some of the patient acceptability interviews. The findings have been disseminated to trial participants, and the results presented at a national user conference.

## Results

### Recruitment, retention, and baseline characteristics

**Fig 1:**
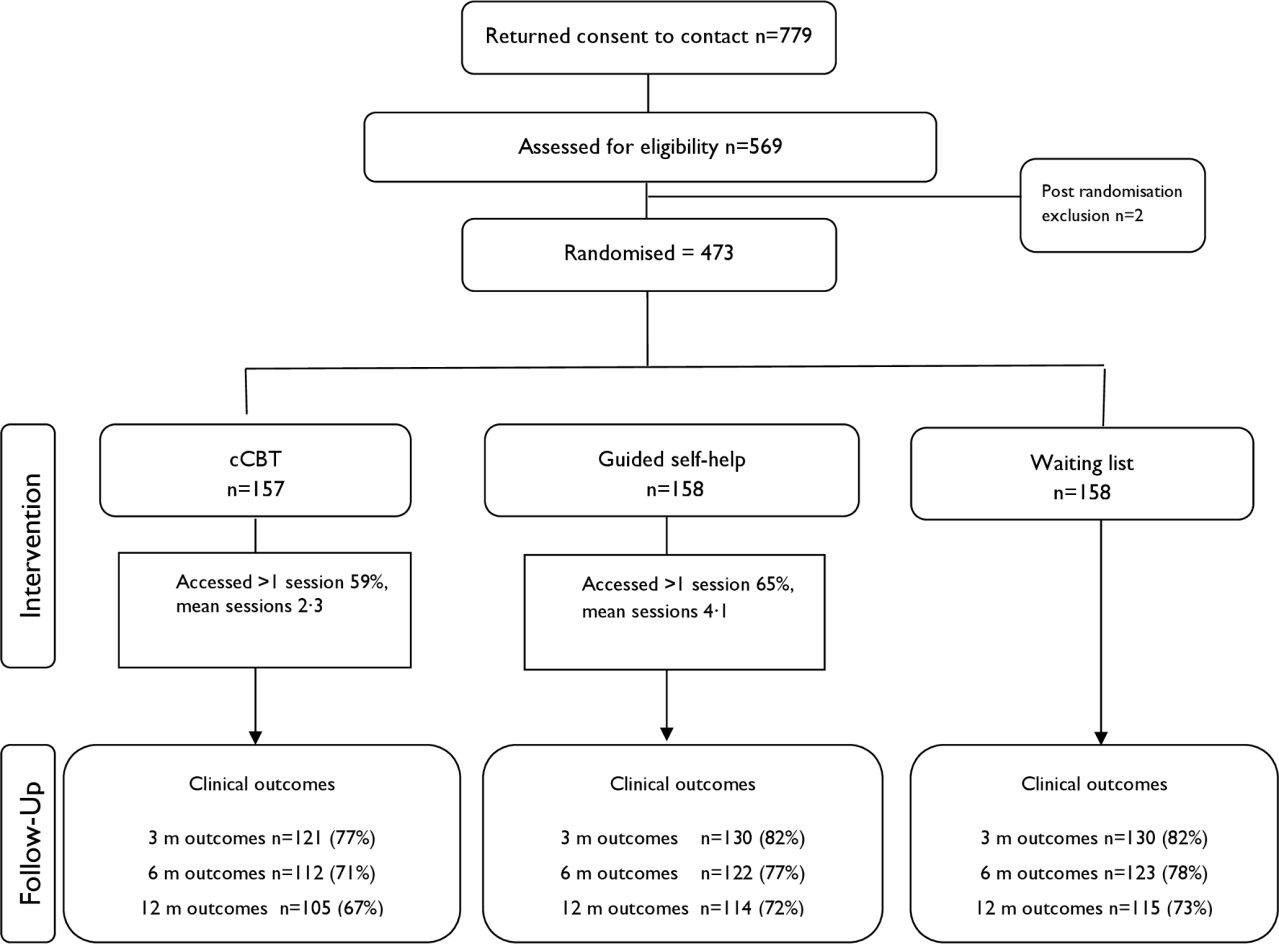
Consolidated Standards of Reporting Trials (CONSORT) flow chart illustrating recruitment. Abbreviations: cCBT, computerised cognitive-behaviour therapy; m, month; QALY, quality-adjusted life year.

We opened recruitment in 15 clinical sites in England between February 2011 and May 2014, with the last follow-up in May 2015. There were 2 postrandomisation exclusions: one aged under 18 years and one at risk of suicide. In total, 473 eligible patients were randomised (see Fig 1). Baseline sociodemographic characteristics are presented in Table 1, with baseline clinical data presented in Table 2. Data were indicative of severe OCD, mild-to-moderate depression, and moderate anxiety. Just over half reported previous professional help with OCD, and around half were using antidepressants. More than half reported OCD for more than 10 years. There were no substantial differences at baseline.

**Table 1:** Baseline sociodemographic variables.

Characteristic	cCBT *n* = 157	Guided self-help *n* = 158	Waiting list *n* = 158
**Age (years): median (range)**	32.0 (18–77)	32.8 (18–72)	33.3 (19–66)
	frequency (%)	frequency (%)	frequency (%)
**Sex**			
Male: *n* (%)	66 (42.0%)	57 (36.1%)	65 (41.1%)
Female: *n* (%)	91 (58.0%)	101 (63.9%)	93 (58.9%)
**Ethnicity**			
White: *n* (%)	145 (92.4%)	154 (97.5%)	149 (94.3%)
Nonwhite: *n* (%)	12 (7.6%)	4 (2.5%)	8 (5.1%)
Missing: *n* (%)	0 (0%)	0 (0%)	1 (0.6%)
**Marital Status**			
Married/living with partner: *n* (%)	84 (53.5%)	81 (51.3%)	85 (53.8%)
Other: *n* (%)	70 (44.6%)	75 (47.4%)	73 (46.2%)
Missing: *n* (%)	3 (1.9%)	2 (1.3%)	0 (0%)
Employment status*			
Employed: *n* (%)	86 (54.4%)	95 (60.1%)	97 (61.4%)
Unemployed and seeking work: *n* (%)	10 (6.3%)	14 (8.9%)	9 (5.7%)
Student: *n* (%)	17 (10.8%)	19 (12.0%)	17 (10.8)
Long-term sick/disabled receiving income support or incapacity benefit: *n* (%)	22 (14.0%)	23 (14.6%)	15 (9.5%)
Homemaker—not actively seeking work: *n* (%)	15 (9.6%)	9 (5.7%)	11 (7.0%)
Not receiving benefits and not actively seeking work: *n* (%)	1 (0.6%)	0 (0%)	1 (0.6%)
Unpaid voluntary work and not actively seeking work: *n* (%)	1 (0.6%)	1 (0.6%)	0 (0%)
Retired: *n* (%)	6 (3.8%)	5 (3.2%)	6 (3.8%)
Missing: *n* (%)	2 (1.3%)	1 (0.6%)	5 (3.2%)
**Receiving statutory sick pay**			
Yes: *n* (%)	8 (5.1%)	8 (5.1%)	11 (7.0%)
No: *n* (%)	144 (91.7%)	146 (92.4%)	138 (87.3%)
Missing: *n* (%)	5 (3.2%)	4 (2.5%)	9 (5.7%)
**Accessed previous OCD help**			
Yes: *n* (%)	76 (48.4%)	86 (54.4%)	72 (45.6%)
No: *n* (%)	80 (51.0%)	71 (44.9%)	85 (53.8%)
Missing: *n* (%)	1 (0.6%)	1 (0.7%)	1 (0.6%)
**Education**			
Below degree level: *n* (%)	107 (68.2%)	110 (69.6%)	112 (70.9%)
Degree level or higher: *n* (%)	45 (28.6%)	43 (27.2%)	40 (25.3%)
Missing: *n* (%)	5 (3.2%)	5 (3.2%)	6 (3.8%)

* *N* and % for all groups do not sum (i.e., to sample size or 100%). This is as a result of some participants indicating more than 1 employment status.

Abbreviations: cCBT, computerised cognitive-behaviour therapy; OCD, obsessive-compulsive disorder.

**Table 2:** Baseline clinical characteristics.

Characteristic	cCBT *n* = 157	Guided self-help *n* = 158	Waiting list *n* = 158
	frequency (%)	frequency (%)	frequency (%)
Current antidepressant medication	82 (52.2%)	81 (51.3%)	80 (50.6%)
**OCD chronicity**			
0–5 years	53 (33.8%)	52 (32.9%)	51 (32.3%)
6–9 years	18 (11.5%)	18 (11.4%)	19 (12.0%)
≥10 years	86 (54.8%)	88 (55.7%)	88 (55.7%)
**Comorbidity (primary diagnosis)**			
Mixed anxiety and depressive disorder	23 (14.6%)	23 (14.6%)	15 (9.5%)
Mild depressive disorder	18 (11.5%)	18 (11.4%)	20 (12.7%)
Moderate depressive disorder	28 (17.8%)	24 (15.2%)	26 (16.5%)
Severe depressive disorder	7 (4.5%)	13 (8.2%)	12 (7.6%)
Generalised anxiety disorder	18 (11.5%)	27 (17.1%)	18 (11.4%)
Specific phobia	10 (6.4%)	6 (3.8%)	6 (3.8%)
Social phobia	2 (1.3%)	1 (1%)	0 (0%)
Agoraphobia	0 (0%)	2 (0.6%)	3 (1.9%)
Panic disorder	2 (1.3%)	0 (0%)	5 (3.2%)
	Mean (SD) *n*	Mean (SD) *n*	Mean (SD) *n*
**Y-BOCS-OR**^a^	25.03 (5.45) 157	25.01 (5.02) 158	25.34 (5.44) 158
Median	25	25	25
Min; Max	13; 38	14; 39	13; 38
Missing	0	0	0
**Y-BOCS-SR**			
Mean (SD)	24.34 (5.1)	24.18 (4.82)	24.20 (4.99)
Median	24	24	24
Min; Max	16;36	16;40	16;38
PHQ-9			
Mean (SD)	11.90 (6.27) 155	11.40 (6.56) 154	11.93 (6.29) 154
Median	12	11.5	12
Min; Max	0; 27	0; 26	0; 26
**GAD-7**			
Mean (SD)	12.90 (5.33)	12.72 (5.56)	12.52 (5.52)
Median	13	14	13
Min; Max	2; 21	1; 21	0; 21
Missing	2	4	4
**CORE-OM**			
Mean (SD)	15.95 (6.27)	15.23 (6.67)	15.79 (6.63)
Median	16	16	16
Min; Max	5; 35	1; 34	1; 33
Missing	3	3	5
**SF36–PCS**			
Mean (SD)	54.39 (11.29)	54.18 (9.57)	54.09 (10.56)
Median	57.36	56.01	57.14
Min; Max	18.04; 71.89	17.59; 70.35	22.21; 72.23
Missing	3	3	5
**SF36–MCS**			
Mean (SD)	32.89 (9.87)	33.86 (11.05)	33.23 (11.71)
Median	32.66	34.33	33.17
Min; Max	11.88; 59.52	7.30; 58.55	10.64; 65.08
Missing	3	3	5
**WSAS**			
Mean (SD)	14.78 (9.85)	15.05 (10.54)	14.74 (9.66)
Median	13	14	13
Min; Max	2; 21	1; 21	0; 21
Missing	2	4	4

^a^ incorporating Y-BOCS-SR if Y-BOCS-OR missing or incomplete. Patients were included in the trial with a Y-BOCS-SR of 16+, so all patients had a Y-BOCS-SR of 16+ at baseline, but some had a Y-BOCS-OR of less than 16.

Abbreviations: cCBT, computerised cognitive-behaviour therapy; CORE-OM, Clinical Outcomes in Routine Evaluation; GAD-7, Generalised Anxiety Disorder 7-item; MCS, Mental Component Summary; OCD, obsessive-compulsive disorder; PCS, Physical Component Summary; SF36, Short Form-36; WSAS, Work and Social Adjustment Scale; Y-BOCS-OR, Yale-Brown Obsessive Compulsive Scale–Observer-Rated; Y-BOCS-SR, Yale-Brown Obsessive Compulsive Scale–Self-Report.

### Treatment fidelity and adherence

**Fig 2:**
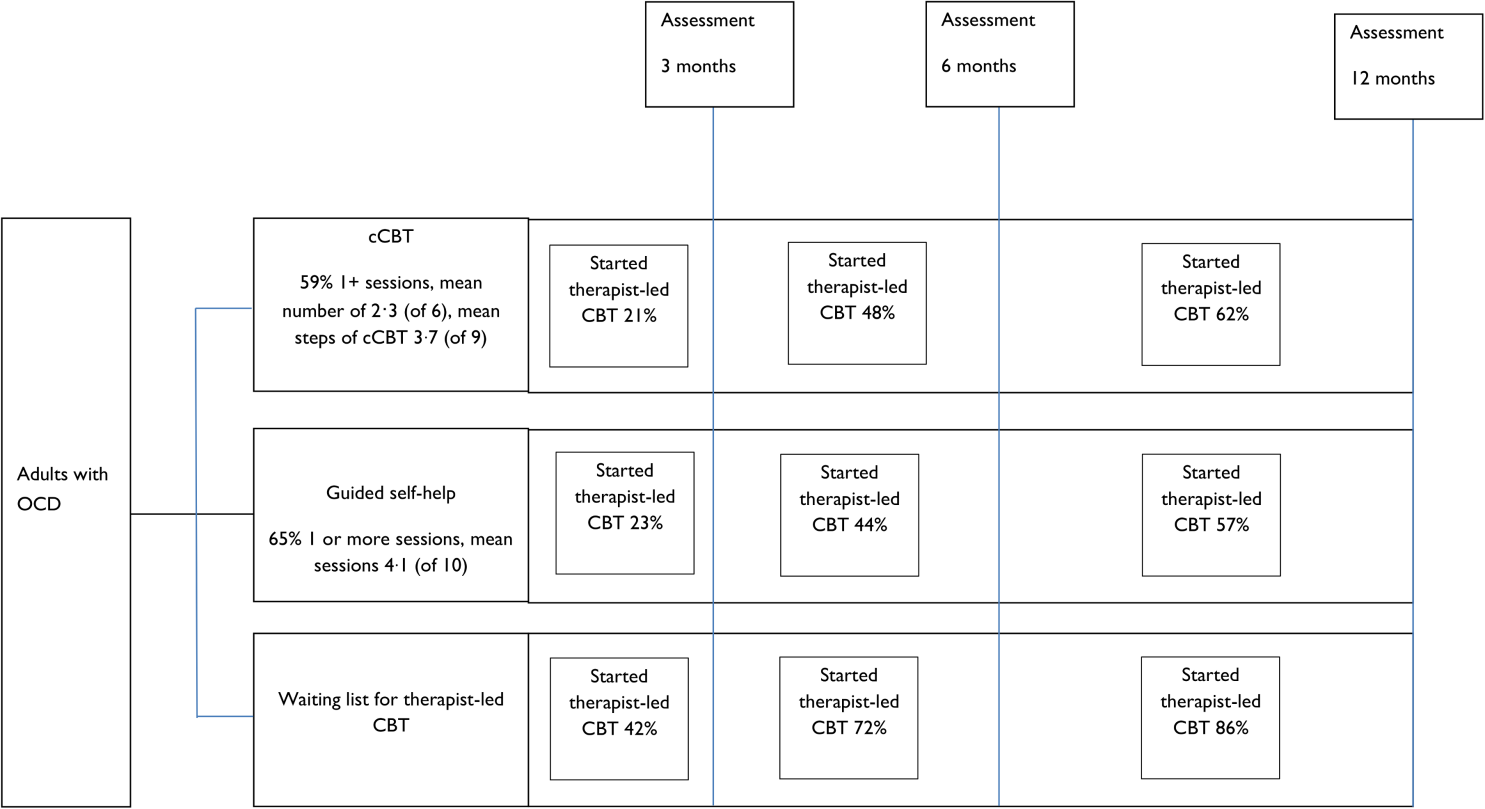
Flow chart illustrating therapist-led CBT uptake. Abbreviations: cCBT, computerised cognitive-behaviour therapy; OCD, obsessive-compulsive disorder.

Patient flow is shown in Figs 1 and 2. Retention rates were 81% at 3 months, 75% at 6 months, and 71% at 12 months and were broadly similar across arms (Fig 1). Contrary to expectation, approximately 29% of patients started to access therapist-led CBT prior to the 3-month assessment (Fig 2). More detailed data on CBT uptake and predictors of uptake are detailed separately (Tables B and C in S1 Appendix).

Fifty-nine percent of participants had at least 1 session with a psychological well-being practitioner in cCBT. The mean number of sessions was 2.3 (SD 2.5), and the average length was 13.4 minutes (93% by telephone). Of the 9 cCBT steps, the mean number completed was 3.7 (SD 3.2). Sixty-five percent of participants had at least 1 session with a psychological well-being practitioner in guided self-help. The mean number of sessions was 4.1 (SD 4.3) over 57 minutes for session 1 and 31 minutes for sessions 2–11 (48% face-to-face, 26% telephone, 22% both, 4% missing). Rates of recording for fidelity assessment were low (26% guided self-help, 17% cCBT). Of the sessions recorded in cCBT, 11 (65%) were rated “good” and 6 (35%) as “excellent.” Of the sessions in guided self-help, 9 (21%) were rated “satisfactory,” 24 (56%) rated “good,” and 10 (23%) “excellent.”

Table 3 gives the summary statistics for the primary (Y-BOCS-OR) and selected secondary outcomes (Y-BOCS-SR, CSQ-8, and EuroQol five dimensions questionnaire [EQ-5D]). Complete outcomes are reported separately (Table D in S1 Appendix).

**Table 3:** Outcome measure summary statistics and intervention effects at 3, 6, and 12 months.

* *	Supported cCBT			Guided self-help			Waiting list			Supported cCBT–Waiting list			Guided self-help–Waiting list			Supported cCBT–Guided self-help		
* *	Mean	SD	*n*	Mean	SD	*n*	Mean	SD	*n*	Adjusted mean difference ^+^	95% CI	*p*	Adjusted mean difference^+^	95% CI	*p*	Adjusted mean difference^+^	95% CI	*p*
Y-BOCS-OR												*** ***			*** ***			*** ***
Baseline	25.03	5.45	157	25.01	5.02	158	25.34	5.44	158									
3 months	21.16	6.89	121	20.19	6.83	130	22.18	6.54	132	−0.71	(−2.12 to 0.70)	0.325	−1.91	(−3.27 to −0.55)	0.006*	1.20	(−0.22 to 2.61)	0.097
6 months	18.96	7.26	112	18.70	7.70	122	20.29	7.27	122	−1.13	(−2.84 to 0.58)	0.195	−1.32	(−3.00 to 0.35)	0.121	0.19	(−1.51 to 1.90)	0.824
12 months	16.14	8.69	105	15.19	8.35	113	17.93	8.07	114	−1.37	(−3.59 to 0.84)	0.224	−2.37	(−4.37 to −0.38)	0.020	1.00	(−1.19 to 3.19)	0.371
Y-BOCS-SR																		
Baseline	24.34	5.10	157	24.18	4.82	158	24.20	4.99	158									
3 months	20.46	7.06	119	19.80	6.90	128	20.88	6.48	127	−0.43	(−1.79 to 0.93)	0.531	−1.31	(−2.65 to 0.04)	0.056	0.87	(−0.49 to 2.23)	0.209
6 months	18.60	7.47	110	18.29	7.78	119	19.34	7.24	118	−0.87	(−2.52 to 0.78)	0.300	−1.17	(−2.87 to 0.53)	0.178	0.30	(−1.42 to 2.02)	0.735
12 months	15.61	8.70	101	15.72	8.11	109	17.38	8.24	107	−1.45	(−3.67 to 0.76)	0.198	−1.52	(−3.54 to 0.49)	0.137	0.07	(−2.01 to 2.16)	0.946
CSQ-8	*** ***	*** ***		*** ***	*** ***		*** ***	*** ***										
3 months	22.41	5.91	92	24.37	5.49	101	22.75	6.06	83	−0.31	(−2.07 to 1.45)	0.732	1.69	(−0.04 to 3.42)	0.055	−2.00	(−3.63 to −0.37)	0.016*
6 months	23.75	5.78	81	24.26	6.29	82	24.64	5.80	77	−0.87	(−2.77 to 1.03)	0.371	−0.31	(−2.23 to 1.61)	0.751	−0.56	(−2.44 to 1.32)	0.561
EQ-5D																		
Baseline	0.67	0.29	155	0.68	0.26	155	0.68	0.26	154									
3 months	0.69	0.31	104	0.73	0.24	117	0.67	0.28	124	0.03	(−0.02 to 0.09)	0.246	0.05	(0.00 to 0.10)	0.042	−0.01	(−0.07 to 0.04)	0.491
6 months	0.71	0.29	94	0.72	0.25	105	0.71	0.25	106	0.00	(−0.06 to 0.06)	0.961	0.00	(−0.05 to 0.07)	0.815	−0.00	(−0.07 to 0.06)	0.864
12 months	0.79	0.27	84	0.73	0.26	100	0.70	0.31	99	0.07	(0.00 to 0.15)	0.041	0.03	(−0.04 to 0.09)	0.449	0.05	(−0.02 to 0.12)	0.179

+ Mean difference adjusted for Y-BOCS-OR, PHQ-9, GAD-7, antidepressant use, gender, and OCD duration (0–5, 6–9, and ≥10 years).

* The significance level is set at 1.67% to adjust for 3 pair-wise comparisons.

Abbreviations: CSQ-8, Client Satisfaction Questionnaire; EQ-5D, EuroQol five dimensions questionnaire; GAD-7, Generalised Anxiety Disorder 7-item; OCD, obsessive-compulsive disorder; PHQ-9, Patient Health Questionnaire; Y-BOCS-OR, Yale-Brown Obsessive Compulsive Scale–Observer-Rated; Y-BOCS-SR, Yale-Brown Obsessive Compulsive Scale–Self-Report.

### Does access to guided self-help or cCBT provide more rapid improvement in OCD symptoms at 3 months compared to a waiting list for therapist-led CBT?

There was no significant benefit of access to cCBT (adjusted mean difference = −0.71, 95% CI −2.12 to 0.70, *p* = 0.325). There was statistically significant benefit of guided self-help (adjusted mean difference = −1.91, 95% CI −3.27 to −0.55, *p* = 0.006), although the effect was less than the prespecified “clinically important difference” of 3 points.

Analyses of secondary outcomes (Tables E, F, and G in S1 Appendix) showed only 1 significant difference, an effect of cCBT on anxiety (adjusted mean difference = −1.50, 95% CI −2.67 to −0.33, *p* = 0.012).

### Does access to guided self-help or cCBT improve patient satisfaction at 3 months compared to a waiting list for therapist-led CBT?

Satisfaction data are shown in Table 3. There were no differences in patient satisfaction among patients receiving cCBT compared to those allocated to a waiting list (adjusted mean difference = −0.31, 95% CI −2.07 to 1.45, *p* = 0.732). Patients receiving guided self-help tended to be more satisfied than those allocated to a waiting list for therapist-led CBT (adjusted mean difference = 1.69, 95% CI −0.04 to 3.42, *p* = 0.055), although the estimate did not reach significance according to the corrected significance level. Patients receiving cCBT were less satisfied than those receiving guided self-help (adjusted mean difference = −2.00, 95% CI −3.63 to −0.37, *p* = 0.016).

### Does access to guided self-help or cCBT prior to therapist-led CBT provide longer-term improvement in OCD symptoms at 12 months compared to therapist-led CBT alone?

There was no significant long-term benefit from access to either guided self-help or cCBT (cCBT adjusted mean difference = −1.37, 95% CI −3.59 to 0.84, *p* = 0.224; guided self-help adjusted mean difference −2.37, 95% CI −4.37 to −0.38, *p* = 0.02; Table 2).

As a post hoc analysis, we tested whether low-intensity interventions were formally noninferior to waiting list for therapist-led CBT at 12 months. A 98.33% confidence interval corresponds to a 1.67% significance level that we have used for hypothesis testing. For the comparison of cCBT against waiting list, the 98.33% confidence interval is −4.07 to 1.33, and for guided self-help against waiting list, it is −4.81 to 0.06. Given that the upper limits are substantially smaller than the prespecified criterion for a clinically important difference (3 points), we conclude that both interventions are noninferior to waiting list at 12 months.

### Does access to guided self-help or cCBT reduce uptake of therapist-led CBT over 12 months?

Therapist-led CBT uptake is shown in Fig 2. Both interventions were associated with significantly lower uptake of therapist-led CBT at 12 months (cCBT adjusted OR = 0.34, 95% CI 0.15 to 0.79 *p* = 0.011; guided self-help adjusted OR = 0.27, 95% CI 0.12 to 0.60 p = 0.001) (Table 4).

**Table 4:** Logistic regression model for CBT uptake at 6 and 12 months.

	Comparison	Adjusted odds ratio	95% CI	*p*
**6 months**				
Group	cCBT versus WL	0.42	(0.24–0.73)	0.002*
	GSH versus WL	0.48	(0.22–1.03)	0.06
	cCBT versus GSH	0.87	(0.42–1.84)	0.718
Baseline outcome measures	Y-BOCS-OR	1.02	(0.97–1.06)	0.514
	GAD-7	0.99	(0.94–1.04)	0.591
	PHQ-9	1.02	(0.98–1.07)	0.271
Antidepressant medication	Yes	0.71	(0.46–1.09)	0.117
Duration of OCD	6–9 years	1.26	(0.60–2.64)	0.552
	10 or more years	0.89	(0.55–1.42)	0.619
Gender	Male	1.12	(0.73–1.73)	0.606
Exponential Function (Constant)		2.14	(0.67–6.82)	0.201
**12 months**				
Group	cCBT versus WL	0.34	(0.15–0.79)	0.011*
	GSH versus WL	0.27	(0.12–0.60)	0.001*
	cCBT versus GSH	1.27	(0.53–3.00)	0.59
Baseline outcome measures	Y-BOCS-OR	1.03	(0.97–1.08)	0.36
	GAD-7	1.03	(0.97–1.08)	0.341
	PHQ-9	0.99	(0.94–1.04)	0.73
Antidepressant medication	Yes	1.02	(0.63–1.67)	0.933
Duration of OCD	6–9 years	2.66	(1.03–6.89)	0.043
	10 or more years	0.99	(0.59–1.67)	0.968
Gender	Male	1.25	(0.76–2.03)	0.395
Exponential Function (Constant)		2.86	(0.76–10.81)	0.121

* Note, the Bonferroni corrected significance level is 1.67%, for 3 pair-wise comparisons.

** Note, results are taken from a logistic regression model and any “effect” should be interpreted as an odds ratio.

Abbreviations: cCBT, computerised cognitive-behaviour therapy; GAD-7, Generalised Anxiety Disorder 7-item; GSH, guided self-help; OCD, obsessive-compulsive disorder; PHQ-9, Patient Health Questionnaire; Y-BOCS-OR, Yale-Brown Obsessive Compulsive Scale–Observer-Rated; WL, waiting list.

Post hoc, we compared intervention use and 12-month OCD outcomes among guided self-help and cCBT patients who did and did not access therapist-led CBT (Table H in S1 Appendix). Although lacking randomisation, the data do not suggest that those who accessed only guided self-help or cCBT demonstrated markedly worse outcomes than those who accessed both a low-intensity intervention and therapist-led CBT (Table I in S1 Appendix).

## Discussion

### Principal outcomes

We assessed the role of 2 low-intensity interventions (guided self-help and cCBT) in OCD. Prior to access to therapist-led CBT, guided self-help demonstrated statistically significant benefits over the waiting list, but the difference did not meet the prespecified criterion for clinical significance. cCBT did not demonstrate significant benefit at the 3- or 12-month follow-up. Access to low-intensity interventions does not provide more rapid symptom relief.

Over 12 months, access to low-intensity interventions prior to therapist-led CBT did not significantly augment the effects of therapist-led CBT on OCD symptoms in the longer term. Rapid access to low-intensity interventions did lead to significant reductions in uptake of therapist-led CBT, which did not compromise patient outcomes at 12 months.

### Strengths and limitations

To our knowledge, we conducted the largest trial of psychological therapy for OCD worldwide. We achieved acceptable levels of retention. When patients were not able to provide the primary clinical outcome using the observer-reported version, we used self-report as a proxy. These different measures show high associations [30,31], with some evidence of lower scores in the self-reported version, but proxy measures were only used in 8% and 11% of cases at 3 and 12 months, with minimal differences in rates of use between arms. In this pragmatic trial, recruitment was over multiple sites and involved a large number of psychological well-being practitioners. This enhances external validity, as delivery was not restricted to a small number of specialised sites or highly selected professionals. However, many psychological well-being practitioners only saw a few patients, which restricted the opportunity to practice their skills. Uptake of the interventions was reasonable (65% guided self-help and 59% cCBT). Collecting detailed data on fidelity proved difficult, but analysis of the data provided some evidence that delivery of guided self-help and cCBT was in line with protocols.

Several issues are worth noting in this pragmatic design. First, we did not mandate a defined waiting time for therapist-led CBT, although the expectation was 3–6 months. In practice, around 40% of patients allocated to a waiting list for therapist-led CBT started to receive some contact with their therapist before 3 months, compared with just over 20% in the guided self-help and cCBT groups. This would reduce differences in outcomes between guided self-help, cCBT, and the waiting list comparator at 3 months, leading to conservative estimates of effect. Still, these data refer to patients receiving any contact with therapist-led CBT, which in most cases would involve an initial session or two, rather than a full “dose” of treatment. Nevertheless, relatively quick access to CBT in the waiting list arm would have reduced short-term benefit associated with the low-intensity interventions. The effects of low-intensity interventions may have been more pronounced at 3 months if CBT was less accessible than in the current trial. The longer-term analyses are less affected, as all patients were expected to receive both a low-intensity intervention (where allocated) and therapist-led CBT over 12 months.

We have shown that uptake of therapist-led CBT was lower in the groups allocated to low-intensity treatment. This could reflect positive outcomes from some aspects of the low-intensity interventions, although our analysis showed that this was largely restricted to patient satisfaction rather than clinical benefits. Even in the absence of significant clinical benefits, providing low-intensity treatments may give patients a sense of support and progress. When combined with natural improvement in symptoms over time (as found in all groups), this may mean that patients do not feel a need for further intensive support or no longer wish to engage with services. However, the numbers of patients attending therapist-led CBT increased in all groups over time. It is possible that, had our follow-up been longer than 12 months, eventual uptake of therapist-led CBT across all groups would be the same.

Secondly, we placed no restrictions on medication use, and in line with most psychological therapy trials in OCD, a proportion of patients were taking medication. Baseline self-reported use of antidepressant treatment is provided in Table 1, showing that about half the patients reported using antidepressants, with no differences between groups. Data on antidepressant use after allocation showed that, over the 12-month period of the trial, self-reported use of antidepressants decreased (cCBT 26%, guided self-help [GSH] 32%, and waiting list 27%) [28]. We do not have details of the nature or quality of antidepressant prescription. Although antidepressants are effective in OCD [3,32], it seems unlikely that such small differences between arms would be a major driver of study outcomes.

Thirdly, there was no attempt to match the level or type of clinician contact across the 2 low-intensity interventions: indeed, the study was specifically designed to test the relative value of 2 different forms of low-intensity intervention. GSH involved both a different delivery format and more clinician contact, so our trial is not a strict comparison of paper and digital interventions. Although increased clinician contact may well enhance acceptability and effectiveness, its additional costs were accounted for in the economic analysis.

Fourth, we did not undertake quality assurance of the therapist-led CBT provided to all patients. As noted above, therapist-led CBT was provided by a range of practitioners in a range of areas and is likely to be reasonably representative of the treatment provided in the National Health Service (NHS) in England, which remains optimal for a pragmatic trial. Formal measurement of quality would have been preferable but logistically complex.

The trial adopted aspects of a stepped care model, whereby patients are offered a low-intensity intervention first, with a proportion progressing to therapist-led CBT. However, unlike true stepped care, there was no regular assessment of outcome, and access to therapist-led CBT was available to all, rather than as part of a defined “stepping” mechanism following nonresponse to treatment or deterioration. Therefore, our analysis does not assess the benefits of low-intensity treatment in a full stepped care model.

### Interpretation of the results in the context of the wider literature

At the initiation of this trial, the evidence base was very limited [11]. While the current trial was being delivered, a number of additional studies were published. One small trial (*n* = 56) used similar interventions to the present study (GSH and supported cCBT) and compared their effects in a group of volunteers recruited through a website. Very large effects were found post-treatment [33]. A second trial (*n* = 86) exploring a minimally supported cCBT intervention again found very large effects in a sample recruited online through a research centre [34]. A third trial randomised 34 patients with OCD to a supported internet-based writing therapy and again found very large effects among a sample recruited via public notices. A long-term follow-up [35] of a previous trial [12] showed enduring effects for cCBT and that “booster” treatments were effective in maintaining gains. Finally, one trial (*n* = 128) explored the effects of unsupported written material about metacognitive therapy in patients with OCD from internet groups, self-help organisations, and clinical facilities and found small benefits [36].

The picture from these trials is more positive about the clinical benefits of “low-intensity” treatments, especially supported cCBT, with most effect sizes over 0.5 and some exceeding 1.0. This contrasts markedly with the modest clinical impacts observed in the current study. There are a number of reasons that could account for these differences. The interventions do vary, although it is not clear that the variation is large enough to account for the large variation in effects. The current sample of patients have more severe symptoms at baseline (Y-BOCS scores of 25, compared to 20–21 in the other studies), although again it is not clear that such modest differences would be expected to lead to such profound variation in impact. Our current study is far larger than the other trials, and some report quite large differences at baseline, which can occur when small numbers of patients are randomised [33,36]. A potentially important issue is the method of recruitment. The vast majority of the patients in the current study were recruited through routine clinical services, whereas a number of the other trials used recruitment through the internet; this may produce a sample with different clinical features and one that is much more amenable to online cCBT interventions. Similar differences in effects between large pragmatic trials in routine services and smaller trials recruiting through the internet have been recently reported in depression [37].

### Implications for service delivery

The trial demonstrated that neither form of low-intensity CBT was responsible for clinically significant improvements in OCD symptoms among patients on the waiting list.

In the absence of any significant clinical benefit over waiting list only, readers may have concerns about reductions in the use of therapist-led CBT at 12 months, as this might reflect the substitution, or delay, of an evidence-based treatment. It may be that access to GSH or cCBT means that patients are put off from engaging in subsequent therapist-led CBT. We found no evidence that lower uptake of therapist-led CBT was associated with worse outcome over 12 months. Concerns that GSH or cCBT inappropriately discourages patients from engaging in subsequent therapist-led CBT are not supported by the wider literature, which shows an increased likelihood of help seeking and greater healthcare use following self-help [38,39]. It is possible that some patients who are offered GSH or cCBT improve so that they do not need subsequent therapist-led CBT or make an informed choice to discontinue therapy sooner rather than later. However, as noted earlier, differences in uptake between arms may have reduced if a longer follow-up had been possible.

Our results raise questions about the targeting of low-intensity interventions. Recruiting from waiting lists identified a sample with severe symptoms and a relatively long history of treatment. Although most showed significant improvements over time (around 8–9 points on the Y-BOCS across groups), the means at 12 months still showed significant symptoms (around 16 points), meaning many would continue to be eligible for the trial. Routine provision of CBT within the NHS in line with clinical guidelines clearly leaves many patients with clinically significant residual symptoms. Low-intensity treatments may be better targeted at a less severely ill group, closer to the onset of their OCD. However, as this patient group is characterised by late presentation to services, the viability of this is unclear.

Neither cCBT nor GSH showed clinically significant benefits at 3 months. Further development of more effective low-intensity interventions may be required. Uptake of the interventions was relatively low, although not abnormally so for a pragmatic trial. Both interventions may benefit from enhancements that might improve motivation or adherence, which might translate to greater clinical benefit.

The clinical results alone do not support an important role for low-intensity interventions in the care pathway for OCD. However, full interpretation of the benefits of low-intensity interventions for OCD also demands consideration of cost-effectivness, using comprehensive assessments of costs, as well as appropriate measures of the impact of these interventions on health-related quality of life and associated utility. We report the results of this analysis separately [28].

## Supporting information

S1 Appendix Supplementary information file. (DOCX)

S1 Text HTA Obsessive Compulsive Treatment Efficacy Trial (OCTET) study protocol v11 02.07.2015. (DOC)

S2 Text Consolidated Standards of Reporting Trials (CONSORT) checklist. (DOC)

## Abbreviations

cCBT: computerised cognitive-behaviour therapy
CBT: cognitive-behaviour therapy
CONSORT: Consolidated Standards of Reporting Trials
CORE-OM: Clinical Outcomes in Routine Evaluation
CSQ-8: Client Satisfaction Questionnaire
DSM-IV: Diagnostic and Statistical Manual of Mental Disorders, Fourth Edition
EQ-5D: EuroQol five dimensions questionnaire
GAD-7: Generalised Anxiety Disorder 7-item
GSH: guided self-help
HTA: Health Technology Assessment
ICC: intracluster correlation
MCS: Mental Component Summary
MHRN: Mental Health Research Network
NHS: National Health Service
OCD: obsessive-compulsive disorder
OCTET: Obsessive Compulsive Treatment Efficacy Trial
OR: odds ratio
PCS: Physical Component Summary
PHQ-9: Patient Health Questionnaire
PWP: Psychological Wellbeing Practitioner
SF-36: Short Form-36
Y-BOCS-OR: Yale-Brown Obsessive Compulsive Scale–Observer-Rated
Y-BOCS-SR: Yale-Brown Obsessive Compulsive Scale–Self-Report
WSAS: Work and Social Adjustment Scale
