# Supplementary material for: Low-intensity cognitive-behaviour therapy interventions for obsessive-compulsive disorder compared to waiting list for therapist-led cognitive-behaviour therapy: 3-arm randomised controlled trial of clinical effectiveness
Source: PLoS Med. 2017 Jun 27;14(6):e1002337. doi: 10.1371/journal.pmed.1002337 (PMC5486961; doi:10.1371/journal.pmed.1002337)
Supplement: S1 Appendix — (DOCX) [file pmed.1002337.s001.docx]

**Table A:** **Psychological Wellbeing Practitioner characteristics**

| **Characteristic** | **Category** | **N=68*** |
| --- | --- | --- |
| Age range in years |  | 24-61 |
| Mean age years (S.D.) |  | 33∙9 (10∙8) |
| Gender (%),Freq∙ (%) | Female  Male | 59 (86∙8)  9 (13∙2) |
| Highest educational qualification Freq. (%) | Undergraduate degree  Post Graduate certificate  Higher Education diploma  Post Graduate diploma  Masters degree  PhD | 12 (17∙6)  30 (44∙1)  1 (1∙5)  14 (20∙6)  9 (13∙2)  1 (1∙5) |
| Length of time in PWP role, Freq. (%) | 6 months – 1 year  1-2 years  2 years – 5 years | 6 (8∙8)  17 (25∙0)  45 (66∙2) |
| Length of time in mental health, Freq. (%) | Up to 1 year  1-5 years  5-10 years  10-20 years | 1 (1∙5)  34 (50∙0)  25 (36∙8)  8 (11∙.8) |
| Received OCD training as part of IAPT training, Freq. (%) | Yes  No | 37 (54∙4)  31 (45∙6) |

*of the 93 psychological wellbeing practitioners who managed patients, 68 returned a demographic questionnaire

**Text A: Uptake of CBT prior to 3-month primary outcome assessment**

Due to changes in service delivery within the services recruited from a number of patients were offered and commenced therapist-led CBT prior to the primary outcome point (Table 4). Those in the waiting list group were significantly more likely to start therapist-led CBT (67, 42%) compared to 21% in cCBT (adjusted OR=0∙36, 95% CI 0∙19 to 0∙68, p= 0∙001) and 23% in guided self-help (adjusted OR=0∙43, 95% CI 0∙22 to 0∙84, p=0∙014). No differences in uptake were identified between cCBT and guided self-help (adjusted OR=0∙84, 95% CI 0∙47 to 1∙51, p=0∙562). No other factors were found to predict therapist-led CBT uptake (Table 5).

**Table B: CBT uptake by group before 3 month assessment**

|  | **cCBT**  **(n=157)** | **Guided self-help**  **(n=158)** | **Waiting list**  **(n=158)** | **Overall**  **(n=473)** |
| --- | --- | --- | --- | --- |
|  | freq. (%) | freq. (%) | freq. (%) | freq. (%) |
| No | 123 (78∙3) | 119 (75∙3) | 92 (58∙2) | 334 (70∙6) |
| Yes | 33 (21∙0) | 37 (23∙4) | 66 (41∙8) | 136 (28∙8) |
| Missing | 1 (0∙6) | 2 (1∙3) | 0 (0∙0) | 3 (0∙6) |

**Table C: Logistic regression model of predictors of high intensity CBT uptake before 3 months**

|  | **Comparison** | **Adj∙ Odds**  **Ratio** | **95%**  **(lower,** | **CI**  **upper)** | **P-value** |
| --- | --- | --- | --- | --- | --- |
|  |  |  |  |  |  |
| Group | cCBT vs WL | 0∙36 | (0∙19, | 0∙68) | 0∙001* |
|  | GSH vs WL | 0∙43 | (0∙22, | 0∙84) | 0∙014 |
|  | cCBT vs GSH | 0∙84 | (0∙47, | 1∙51) | 0∙562 |
|  |  |  |  |  |  |
| Baseline outcome measures | YBOC-OR | 1∙02 | (0∙97, | 1∙07) | 0∙462 |
|  | GAD-7 | 1∙01 | (0∙96, | 1∙06) | 0∙828 |
|  | PHQ-9 | 0∙99 | (0∙95, | 1∙04) | 0∙789 |
|  |  |  |  |  |  |
| Anti-depressant medication | Yes | 1∙02 | (0∙66, | 1∙57) | 0∙931 |
|  |  |  |  |  |  |
| Duration of OCD | 6 - 9 years | 1∙12 | (0∙55, | 2∙27) | 0∙756 |
|  | 10 or more years | 0∙89 | (0∙55, | 1∙42) | 0∙606 |
|  |  |  |  |  |  |
| Gender | Male | 1∙21 | (0∙78, | 1∙88) | 0∙395 |
|  |  |  |  |  |  |
| Exp (Cons)+ |  | 0∙44 | (0∙14, | 1∙36) | 0∙157 |

* Significance level is set at 1∙67% to adjust for 3 pair-wise comparisons

+ Exponent of logistic regression model constant

**Table D: 3, 6 and 12-month outcomes for the primary outcome measure (Y-BOCS-OR) and self-report version (Y-BOCS-SR)**

|  | **cCBT** | **Guided self-help** | **Waiting List** | **cCBT - Waiting List** | **Guided self-help –Waiting List** | **cCBT- guided self-help** |
| --- | --- | --- | --- | --- | --- | --- |
| **Y-BOCS-OR** | **Mean (SD) n** | **Mean (SD) n** | **Mean (SD) n** | **Adj. Mean Diff^+^ (95% CI) p*** | **Adj. Mean Diff^+^ (95% CI) p*** | **Adj. Mean Diff^+^ (95% CI) p*** |
| Baseline | 25∙03 (5∙45) 157 | 25∙01 (5∙02) 158 | 25∙34 (5∙44) 158 |  |  |  |
| 3 Months | 21∙16 (6∙89) 121 | 20∙19 (6∙83) 130 | 22∙18 (6∙54) 132 | -0∙71 (-2∙12, 0∙70) 0∙325 | -1∙91 (-3∙27, -0∙55) 0∙006* | 1∙2 (-0∙22, 2∙61) 0∙097 |
| 6 Months | 18∙96 (7∙26) 112 | 18∙70 (7∙7) 122 | 20∙29 (7∙27) 122 | -1∙13 (-2∙84, 0∙58) 0∙195 | -1∙32 (-3∙00, 0∙35) 0∙121 | 0∙19 (-1∙51, 1∙90) 0∙824 |
| 12 months | 16∙14 (8∙69) 105 | 15∙19 (8∙35) 113 | 17∙93 (8∙07) 114 | -1∙37 (-3∙59, 0∙84) 0∙224 | -2∙37 (-4∙37, -0∙38) 0∙020 | 1∙00 (-1∙19, 3∙19) 0∙371 |
| **Y-BOCS-SR** | **Mean (SD) n** | **Mean (SD) n** | **Mean (SD) n** | **Adj. Mean Diff^+^ (95% CI) p*** | **Adj. Mean Diff^+^ (95% CI) p*** | **Adj. Mean Diff^+^ (95% CI) p*** |
| Baseline | 24∙34 (5∙1) 157 | 24∙18 (4∙82) 158 | 24∙20 (4∙99) 158 |  |  |  |
| 3 Months | 20∙46 (7∙06) 119 | 19∙8 (6∙9) 128 | 20∙88 (6∙48) 127 | -0∙43 (-1∙79, 0∙93) 0∙531 | -1∙31 (-2∙65, 0∙04) 0∙056 | 0∙87 (-0∙49, 2∙23) 0∙209 |
| 6 Months | 18∙60 (7∙47) 110 | 18∙29 (7∙78) 119 | 19∙34 (7∙24) 118 | -0∙87 (-2∙52, 0∙78) 0∙3 | -1∙17 (-2∙87, 0∙53) 0∙178 | 0∙3 (-1∙42, 2∙02)0∙735 |
| 12 months | 15∙61 (8∙7) 101 | 15∙72 (8∙11) 109 | 17∙38 (8∙24) 107 | -1∙45 (-3∙67, 0∙76) 0∙198 | -1∙52 (-3∙54, 0∙49) 0∙137 | 0∙07 (-2∙01, 2∙16) 0∙946 |

+ Mean difference adjusted for Y-BOCS-OR, PHQ-9, GAD-7,anti-depressant use, Gender, OCD duration ( 0-5, 6-9, ≥10 years)∙ Statistical inference based on all subjects with outcome data * Significance level is set at 1∙67% to adjust for 3 pair-wise comparisons

**Table E:** **3, 6 and 12 month outcomes for the secondary outcome measures**

|  | **cCBT** | **Guided self-help** | **Waiting List** | **cCBT - Waiting List** | **Guided self-help –Waiting List** | **cCBT- guided self-help** |
| --- | --- | --- | --- | --- | --- | --- |
| **SF36v2 PCS** | **Mean (SD) n** | **Mean (SD) n** | **Mean (SD) n** | **Adj. Mean Diff^+^ (95% CI) p*** | **Adj. Mean Diff^+^ (95% CI) p*** | **Adj. Mean Diff^+^ (95% CI) p*** |
| Baseline | 54∙4 (11∙3) 154 | 54∙2 (9∙6) 155 | 54∙1 (10∙6) 153 |  |  |  |
| 3 Months | 53∙6 (10∙8) 104 | 53∙5 (10∙0) 117 | 53∙9 (10∙0) 123 | 0∙14 (-1∙58 ,1∙87) 0∙870 | -0∙38 (-2∙22, 1∙46) 0∙686 | 0∙52 (-1∙51 ,2∙56) 0∙614 |
| 6 Months | 53∙4 (10∙8) 91 | 53∙9 (10∙0) 107 | 51∙8 (10∙7) 107 | 0∙64 (-1∙46 ,2∙75) 0∙550 | 1∙36 (-0∙43 ,3∙15) 0∙140 | -0∙71 (-2∙74 ,1∙31) 0∙490 |
| 12 months | 54∙7 (9∙7) 84 | 53∙4 (10∙0) 97 | 53∙1 (10∙9) 98 | 0∙69 (-1∙20, 2∙57) 0∙474 | -0∙37 (-2∙18, 1∙43) 0∙685 | 1∙06 (-0∙82, 2∙94) 0∙269 |
| **SF36v2 MCS** | **Mean (SD) n** | **Mean (SD) n** | **Mean (SD) n** | **Adj. Mean Diff^+^ (95% CI) p*** | **Adj. Mean Diff^+^ (95% CI) p*** | **Adj. Mean Diff^+^ (95% CI) p*** |
| Baseline | 32∙9(9∙9) 154 | 33∙9 (11∙1) 155 | 33∙2 (11∙7) 153 |  |  |  |
| 3 Months | 37∙0 (11∙7) 104 | 36∙3 (12∙1) 117 | 35∙6 (11∙5) 123 | 1∙48 (-1∙12 ,4∙09) 0∙264 | 0∙46 (-1∙82 ,2∙73) 0∙694 | 1∙03 (-1∙57 ,3∙62) 0∙438 |
| 6 Months | 38∙9 (10∙8) 91 | 37∙3 (12∙6) 107 | 38∙1 (11∙9) 107 | 0∙87 (-1∙90, 3∙63) 0∙540 | -1∙66 (-4∙36 ,1∙04) 0∙230 | 2∙53 (-0∙25, 5∙30) 0∙070 |
| 12 months | 43∙0 (11∙6) 84 | 40∙7 (12∙7) 97 | 40∙0 (11∙4) 98 | 2∙29 (-0∙94, 5∙51) 0∙165 | 0∙13 (-2∙79, 3∙05) 0∙932 | 2∙16 (-1∙11, 5∙43) 0∙195 |

| **PHQ-9** | **Mean (SD) n** | **Mean (SD) n** | **Mean (SD) n** | **Adj. Mean Diff^+^ (95% CI) p*** | **Adj. Mean Diff^+^ (95% CI) p*** | **Adj. Mean Diff^+^ (95% CI) p*** |
| --- | --- | --- | --- | --- | --- | --- |
| Baseline | 11∙9 (6∙3) 157 | 11∙4 (6∙6) 158 | 11∙9 (6∙3) 158 |  |  |  |
| 3 Months | 9∙3 (6∙5) 105 | 9∙1 (6∙0) 118 | 9∙6 (6∙0) 124 | -0∙48 (-1∙66 ,0∙70) 0∙427 | -0∙34 (-1∙48 ,0∙81) 0∙565 | -0∙14 (-1∙33 ,1∙05) 0∙815 |
| 6 Months | 8∙3 (6∙1) 96 | 8∙9 (5∙8) 107 | 8∙5 (5∙9) 106 | -0∙09 (-1∙42 ,1∙24) 0∙890 | 0∙73 (-0∙53 ,1∙99) 0∙260 | -0∙82 (-2∙10 ,0∙46) 0∙210 |
| 12 months | 6∙4 (5∙9) 86 | 7∙1 (5∙9) 99 | 7∙7 (6∙1) 98 | -1∙35 (-2∙96, 0∙25) 0∙098 | -0∙41 (-1∙78, 0∙96) 0∙557 | -0∙94 (-2∙50, 0∙62) 0∙236 |
| **GAD-7** | **Mean (SD) n** | **Mean (SD) n** | **Mean (SD) n** | **Adj. Mean Diff^+^ (95% CI) p*** | **Adj. Mean Diff^+^ (95% CI) p*** | **Adj. Mean Diff^+^ (95% CI) p*** |
| Baseline | 12∙9 (5∙3) 155 | 12∙7 (5∙6) 154 | 12∙5 (5∙5) 154 |  |  |  |
| 3 Months | 9∙9 (5∙9) 104 | 10∙6 (5∙7) 115 | 11∙2 (5∙8) 124 | -1∙50 (-2∙67, -0∙33) 0∙012* | -0∙77 (-1∙91, 0∙37) 0∙186 | -0∙73 (-1∙97, 0∙50) 0∙245 |
| 6 Months | 9∙4 (5∙9) 94 | 9∙4 (5∙6) 107 | 9∙4 (5∙9) 107 | -0∙24 (-1∙63, 1∙15) 0∙730 | 0∙15(-1∙19, 1∙49) 0∙830 | -0∙39 (-1∙78, 0∙99) 0∙580 |
| 12 months | 7∙8 (6∙0) 84 | 8∙0 (5∙8) 100 | 9∙0 (6∙0) 98 | -1∙04 (-2∙64, 0∙55) 0∙199 | -0∙89 (-2∙43, 0∙64) 0∙253 | -0∙15 (-1∙70, 1∙40) 0∙849 |
| **CORE-OM** | **Mean (SD) n** | **Mean (SD) n** | **Mean (SD) n** | **Adj. Mean Diff^+^ (95% CI) p*** | **Adj. Mean Diff^+^ (95% CI) p*** | **Adj. Mean Diff^+^ (95% CI) p*** |
| Baseline | 16∙0 (6∙3) 154 | 15∙2 (6∙7) 155 | 15∙8 (6∙6) 153 |  |  |  |
| 3 Months | 12∙9 (6∙9) 104 | 13∙0 (6∙6) 116 | 13∙5 (6∙7) 124 | -0∙38 (-1∙61, 0∙85) 0∙550 | -0∙20 (-1∙38, 0∙98) 0∙734 | -0∙17 (-1∙48, 1∙14) 0∙797 |
| 6 Months | 11∙8 (6∙8) 90 | 12∙8 (6∙9) 106 | 12∙3 (6∙7) 107 | -0∙16 (-1∙61, 1∙28) 0∙830 | 1∙30 (-0∙13, 2∙74) 0∙080 | -1∙47 (-2∙93, -0∙01) 0∙050 |
| 12 months | 9∙7 (7∙1) 82 | 10∙7 (7∙4) 97 | 11∙3 (7∙4) 98 | -0∙81 (-2∙65, 1∙02) 0∙385 | -0∙16 (-1∙92, 1∙60) 0∙857 | -0∙65 (-2∙47, 1∙16) 0∙482 |

| **WSAS** | **Mean (SD) n** | **Mean (SD) n** | **Mean (SD) n** | **Adj. Mean Diff^+^ (95% CI) p*** | **Adj. Mean Diff^+^ (95% CI) p*** | **Adj. Mean Diff^+^ (95% CI) p*** |
| --- | --- | --- | --- | --- | --- | --- |
| Baseline | 14∙78 (9∙85) 153 | 15∙05 (10∙54) 154 | 14∙74 (9∙66) 154 |  |  |  |
| 3 Months | 12∙98 (11∙01) 104 | 13∙70 (10∙41) 117 | 13∙42 (9∙65) 123 | -0∙55 (-2∙35, 1∙26) 0∙554 | 0∙02 (-1∙17, 1∙75) 0∙985 | -0∙56 (-2∙38, 1∙25) 0∙544 |
| 6 Months | 12∙46 (11∙09) 94 | 13∙11 (10∙53) 106 | 12∙48 (9∙83) 107 | -0∙06 (-2∙24, 2∙12) 0∙956 | 0∙60 (-1∙56, 2∙76) 0∙587 | -0∙66 (-2∙87,1∙55) 0∙557 |
| 12 months | 10∙65 (10∙34) 84 | 11∙94 (11∙11) 99 | 12∙37 (10∙69) 98 | -0∙39 (-3∙16, 2∙38) 0∙782 | -0∙31 (-2∙67, 2∙06) 0∙798 | -0∙08 (-2∙82, 2∙65) 0∙952 |

+ Mean difference adjusted *for Y-BOCS-OR, PHQ-9, GAD-7, Anti-depressant use, Gender, OCD duration (0-5, 6-9,≥10 years)∙*

Statistical inference based on all subjects with outcome data

* Significance level is set at 1∙67% to adjust for 3 pair-wise comparisons

**Table F: Comparison of IAPT Employment Status at follow-up**

|  | **cCBT**  **n=98** | **Guided self help**  **n=109** | **Waiting List**  **n=109** |
| --- | --- | --- | --- |
|  |  |  |  |
| Employed: n (%) | 45 (45∙9%) | 63 (57∙8%) | 58 (53∙2%) |
| Unemployed and seeking work: n (%) | 8 (8∙2%) | 3 (2∙8%) | 5 (4∙6%) |
| Student: n (%) | 6 (6∙1%) | 11 (10∙1%) | 7 (6∙4%) |
| Long term sick/disabled receiving income support or incapacity benefit: n (%) | 8 (8∙2%) | 16 (14∙7%) | 14 (12∙8%) |
| Homemaker – not actively seeking work: n (%) | 10 (10∙2%) | 5 (4∙6%) | 7 (6∙4%) |
| Not receiving benefits and not actively seeking work: n (%) | 0 (0%) | 1 (0∙9%) | 1 (0∙9%) |
| Unpaid voluntary work and not actively seeking work: n (%) | 3 (3∙1%) | 0 (0%) | 2 (1∙8%) |
| Retired: n (%) | 5 (5∙1%) | 4 (3∙7%) | 6 (5∙5%) |

*N and % for all groups do not sum correctly (i∙e∙ to sample size or 100%). This is as a result of this being a participant self-completed measure with some participants indicating more than one employment status.*

**Table G:** **Descriptive data (Pathway Questionnaire) on CBT uptake at 6 and 12-months**

|  | **cCBT**  **(n=157)** | **Guided Self- Help**  **(n=158)** | **Waiting list**  **(n=158)** | **Overall**  **(n=473)** |
| --- | --- | --- | --- | --- |
|  | freq. (%) | freq. (%) | freq. (%) | freq. (%) |
| 6 months |  |  |  |  |
| No | 68 (43∙3) | 72 (45∙6) | 38 (24∙1) | 178 (37∙6) |
| Yes | 76 (48∙4) | 69 (43∙7) | 113 (71∙5) | 258 (54∙6) |
| Missing | 13 (8∙3) | 17 (10∙8) | 7 (4∙4) | 37 (7∙8) |
| 12 months |  |  |  |  |
| No | 44 (28∙0) | 50 (31∙7) | 17 (10∙8) | 111 (23∙5) |
| Yes | 98 (62∙4) | 90 (56∙9) | 136 (86∙1) | 324 (68∙5) |
| Missing | 15 (9∙6) | 18 (11∙4) | 5 (3∙2) | 38 (8∙0) |

**Table H: Number of supported cCBT and guided self-help sessions by uptake of high intensity CBT at 12 months**

|  | **High Intensity CBT received** | **Mean** | **(S.D.)** | **n** |
| --- | --- | --- | --- | --- |
| cCBT | No | 3∙33 | 2∙55 | 30 |
|  | Yes | 3∙95 | 2∙44 | 65 |
| Guided self-help | No | 5∙41 | 4∙63 | 41 |
|  | Yes | 5∙1 | 3∙96 | 70 |

**Table I: Y-BOCS-OR summaries by CBT uptake at 12 months**

| **Y-BOCS-OR** |  |  | **Mean** | **(S.D.)** | **n** |
| --- | --- | --- | --- | --- | --- |
| **(Proxy inc)** | |  |  |  |  |
|  | Allocation | CBT received |  |  |  |
| Baseline |  |  |  |  |  |
|  | cCBT | No | 24∙05 | 5∙66 | 44 |
|  |  | Yes | 25∙16 | 5∙25 | 98 |
|  | GSH | No | 24∙70 | 5∙21 | 50 |
|  |  | Yes | 25∙43 | 4∙71 | 90 |
|  | WL | No | 23∙82 | 4∙65 | 17 |
|  |  | Yes | 25∙59 | 5∙59 | 136 |
| 3 Months |  |  |  |  |  |
|  | cCBT | No | 19∙42 | 7∙60 | 33 |
|  |  | Yes | 21∙63 | 6∙62 | 83 |
|  | GSH | No | 20∙19 | 7∙50 | 42 |
|  |  | Yes | 20∙59 | 6∙26 | 79 |
|  | WL | No | 22∙13 | 6∙08 | 15 |
|  |  | Yes | 22∙17 | 6∙66 | 114 |
| 6 Months |  |  |  |  |  |
|  | cCBT | No | 18∙34 | 7∙29 | 29 |
|  |  | Yes | 19∙00 | 7∙36 | 78 |
|  | GSH | No | 18∙51 | 8∙26 | 41 |
|  |  | Yes | 18∙96 | 7∙58 | 75 |
|  | WL | No | 21∙67 | 6∙36 | 12 |
|  |  | Yes | 20∙11 | 7∙41 | 107 |
| 12 Months |  |  |  |  |  |
|  | cCBT | No | 16∙81 | 8∙71 | 31 |
|  |  | Yes | 15∙67 | 8∙69 | 72 |
|  | GSH | No | 16∙37 | 8∙45 | 38 |
|  |  | Yes | 14∙59 | 8∙29 | 75 |
|  | WL | No | 20∙07 | 7∙01 | 14 |
|  |  | Yes | 17∙59 | 8∙23 | 99 |
