# Supplementary material for: Low-intensity cognitive-behaviour therapy interventions for obsessive-compulsive disorder compared to waiting list for therapist-led cognitive-behaviour therapy: 3-arm randomised controlled trial of clinical effectiveness
Source: PLoS Med. 2017 Jun 27;14(6):e1002337. doi: 10.1371/journal.pmed.1002337 (PMC5486961; doi:10.1371/journal.pmed.1002337)
Supplement: S1 Text — (DOC) [file pmed.1002337.s002.doc]

**1. Project title:** 09/81/01 Obsessive Compulsive Treatment Efficacy Trial (OCTET)

**2. Summary**

Obsessive compulsive disorder (OCD) is a common problem affecting 13% of the population. OCD makes people anxious and unhappy, interferes with everyday activities such as working or looking after relatives and it costs patients, their families and the country a lot of money. OCD rarely improves without treatment. New treatment guidelines were developed by experts in 2005. These guidelines recommend that people with OCD receive a form of psychological help, or talking treatment, called cognitive behavioural therapy (CBT). The guidelines suggest that this CBT might be delivered as a selfhelp approach. In selfhelp, CBT is delivered through a book or computer programme, with some guidance from a mental health professional. Guidance can be delivered over the telephone, by email or face to face.

However, we still do not know which method of selfhelp is most useful, or how useful they are compared to usual care. Our proposed study will test 2 different self help treatments for OCD. These are 1) computerised CBT (cCBT) using an internet delivered OCD treatment package called OCFighter, with telephone or face-to-face support from a mental health professional; and 2) a selfhelp book which helps people to use CBT combined with face to face or telephone support, from a mental health professional. This treatment is called guided self help. Both treatments will be delivered over a 12week period. We want to see if the new treatments (cCBT and guided self help) are effective in the short and longer term. Our study will also find out how satisfied people are with the treatments and which they prefer. Finally, we will calculate the costs of each treatment to OCD sufferers, their families and the NHS.

**3. Planned Investigation**

**3.1 Research objectives:**

Our study aims to:

1. Identify and confirm estimated recruitment rates for an OCD treatment trial via an internal pilot phase aimed at evaluating recruitment rates and primary outcome point.

2. Proceed seamlessly to a full RCT (if recruitment is successful in the pilot phase) to determine:

(a) The clinical and cost effectiveness of two self-managed CBT interventions (cCBT and bibliotherapy) compared to a CBT waiting list in the management of OCD patients in the short term at 3 and 6 month follow up

(b) The clinical and cost effectiveness of self managed therapies plus conventional CBT compared to waiting list plus conventional CBT at 12 month follow up

3. Determine patient compliance and patient and health professional acceptability of the two self managed therapy packages (cCBT & GSH).

**3.2 Existing research:**

Obsessive compulsive disorder (OCD) is a chronic and disabling mental health condition ranked as the 10th leading cause of disability by the World Health Organization [3]. The obsessions and compulsions that characterise this disorder lead to marked distress, are time consuming and significantly interfere with an individual's functioning. The UK prevalence of OCD is 1.1-3.0% [4,5], and unless adequately treated it remains a chronic condition. The NICE guidelines for OCD [6] specify recommendations for the treatment and management of OCD using a stepped care approach. Steps 3-6 of this model recommend treatment options for people with OCD that range from low intensity, primary care-led guided self help to more intensive psychological and pharmacological interventions. Cognitive Behavioral Therapy (CBT) including Exposure and Response Prevention (ERP) is the recommended psychological treatment.

Low intensity interventions are defined as less than 10 hours of therapist time and include CBT (including ERP) with self-managed materials and brief individual CBT by telephone. There is insufficient evidence of the efficacy, cost effectiveness and acceptability of self managed therapy interventions for OCD. Two uncontrolled studies of self help materials with guidance from a therapist [7,8] demonstrate promising results. A trial using CBT delivered by telephone compared with face to face delivery showed similar clinical gains and patient satisfaction [1]. An uncontrolled study [9] which delivered CBT by telephone with self help materials found similar effect sizes as face to face trials.

NICE did not recommend cCBT as a treatment. The commissioning brief requested a trial of self managed therapies with specific reference to cCBT and bibliotherapy. A recent systematic review [10] of cCBT for OCD found only 4 studies, all using the software programme OCfighter (previously known as BT Steps). Results showed significantly better outcomes and less attrition with scheduled compared to unscheduled telephone support. The conclusion of the review found OCfighter to be as good as standard therapist delivered CBT in reducing time spent in rituals and obsessions and in improving work and social functioning. Overall, standard therapist-delivered CBT was more effective than OCfighter but not for those who actually started as opposed to those who failed to begin self exposure therapy.

A key limitation of this work is that all OCfighter evaluations have been conducted by the commercial company who developed the programme. Further, this programme was originally delivered with an interactive voice response (IVR) and workbook. A more recent version, which has not yet been evaluated in an RCT, comprises a web-based platform in conjunction with brief support via telephone, face-to-face or email contact with a mental health worker. A cost effectiveness analysis has been completed [11] with the original BT steps programme but this was not independent from the developers of the commercially produced package. No studies have compared cCBT to bibliotherapy, nor do we know the numbers of people who will not improve with self-managed packages and who will require more intensive CBT. Thus whilst there is some preliminary evidence that self managed therapy packages for OCD can be effective a more robust evidence base of efficacy, cost effectiveness and acceptability framed within UK NHS services is required.

**3.3 Research Methods:**

The HTA brief calls for an evaluation of self-managed therapy packages for Obsessive Compulsive Disorder (OCD) compared to treatment as usual (i.e. waiting for therapist-based cognitive behaviour therapy). The brief highlights the need for a preliminary pilot phase to ensure recruitment targets can be reached. We propose to conduct i) a pilot phase to determine the feasibility of recruiting to a larger trial. If successful in recruitment in the pilot phase we will proceed seamlessly to ii) a full 3-arm trial as stipulated in the brief. We aim to use a commercially available cCBT package (OC-fighter) delivered via the internet and an existing CBT bibliotherapy package. Both of these technologies have the advantage of being accessible to participants at a time and place convenient to them. To achieve our objectives and meet the commissioning brief we have designed a 2-phase study.

**3.3.1 Phase 1: Pilot Phase**:

The commissioning brief recommended a preliminary phase incorporating a pilot phase to ensure that we are able to recruit the numbers needed for a fully powered RCT. Recruitment to mental health trials is routinely problematic and no trial of OCD has as yet been conducted using the numbers that we propose for a fully powered trial. These potential recruitment difficulties may be further compounded by the UK Increasing Access to Psychological Therapies (IAPT) agenda. The additional resources associated with IAPT could mean that waiting lists for conventional high intensity therapist led CBT decrease. However, the true impact of IAPT on waiting lists remains unknown at the national level and any noticeable change in waiting lists may be partially offset by the 30% rise in mental health referrals following the current economic downturn. Therefore an internal pilot phase is needed to both assess recruitment rates and determine the actual length of time potential participants remain on a waiting list before proceeding to a full trial. Although we have explored the length of current CBT list it is crucial that we accurately assess future waiting times during the pilot phase at each of our study sites in order that we can determine the most accurate short-term follow-up assessment point

Three key questions will be answered via the pilot phase

1. Is it feasible to recruit the numbers required for a fully powered RCT in the designated time available?
2. Do participants remain on a CBT waiting list for a sufficient length of time (i.e. at least 3 months) to conduct an evaluation of the short-term clinical and cost effectiveness of self managed therapies?
3. Is it feasible to retain the proposed 6-month outcome assessment for short term clinical effectiveness in addition to a 3-month follow-up assessment?

We will seek to recruit 153 patients who meet our inclusion criteria (section 3.6) and randomise to i) cCBT, ii) guided self help (bibliotherapy) or iii) CBT waiting list. We will recruit from all 4 of our clinical sites (Manchester, York, Sheffield and East Anglia). Our full trial procedures will be mirrored in the pilot study. Within the pilot phase we will also modify the Adult Service Use Schedule (AD-SUS) to ensure it adequately captures resources appropriate to OCD, as described in Section 3.11 below.

***Criteria for successful recruitment:*** The success of our pilot will be based on reviewing a number of key indicators, one of which is recruitment. We aim to recruit approximately 153 participants in the pilot study and should we achieve or surpass this target then we will accept that we have met our recruitment target. Should we have a significant shortfall however, we will investigate and report the reasons for this to assess whether or not these are transient (e.g. a delay in research governance) that will not lead to a recruitment reduction overall or permanent (e.g. a lack of sufficient consenting patients). Depending on the reasons we will make a recommendation to our independent TSC, and through them to the HTA who will make the decision as to whether the trial should proceed.

***Procedure to proceed to full trial:*** If we are successful in meeting our criteria for recruitment at the end of our pilot study we will proceed to a full trial with permission from the HTA.

**3.3.2 Phase 2: Full RCT:**

If successful in recruiting to target in the pilot study (153 patients in 9 months) and with agreement from the HTA we will proceed seamlessly to a full RCT including a concurrent economic evaluation. This will be a multi-centre fully randomised controlled trial to evaluate 2 self managed packages (cCBT and bibliotherapy) compared to waiting list prior to therapist-based CBT.

The key objectives of our study are to determine:

1. The clinical and cost effectiveness of two self-managed CBT interventions (cCBT and bibliotherapy) compared to a CBT waiting list in the management of OCD patients in the short term at 3 month follow up
2. The clinical and cost effectiveness of self managed therapies plus conventional CBT compared to waiting list plus conventional CBT at 6 and 12 month follow up
3. Determine patient compliance and patient and health professional acceptability of the two self managed therapy packages (cCBT & GSH).

We will randomise patients to i) cCBT, ii) bibliotherapy (guided self help) or iii) CBT waiting list.

The primary outcome will be OCD symptoms as measured by the Yale Brown Obsessive Compulsive Checklist YBOCS [12] observer-report version. Secondary outcomes will include self-reported health-related quality of life (SF-36), self-reported OCD symptoms (YBOCs self-rated), generic mental health (CORE-OM), depression (PHQ9), anxiety (GAD-7), functioning (WSA), health-related quality of life (EQ5D), employment status (IAPT Employment Status questions A13 –A15), patient satisfaction (CSQ), attachment (RSQ), Perceived Criticism (PCS), expressed emotion (FEICS), patient progression through mental health service (Pathway questionnaire), patient and therapist acceptability (qualitative interviews), and % patients not improved or partially improved and requiring more intensive CBT. Participants will additionally be asked to complete a demographic questionnaire at baseline.

Our preferred primary outcome point was at 6-months follow-up. However, the results of our pilot phase determined identified that this will need to be limited to 3-months as the vast majority of patients had commenced CBT treatment by this point. The primary outcome point will thus reflect CBT waiting lists at the time of study, and as such will enable us to determine an estimate of short term effectiveness of the self-managed therapy packages compared to a CBT waiting list control. This design will not require patients within the trial to have restricted access to treatment beyond that already associated with resource limitations at each site.

At 6 and 12 months follow–up we would expect participants randomised to the waiting list to have accessed conventional CBT and participants randomised to one of the self managed packages (either cCBT or bibliotherapy) will have either remained on the waiting list and accessed conventional CBT or improved sufficiently with the self managed package that they no longer require conventional CBT. The 6 and 12 month follow-up points will enable us to provide a pragmatic demonstration of the clinical and cost-effectiveness of self-managed therapy plus or minus conventional CBT versus waiting-list followed by conventional CBT in the longer term. We will be able to explore a number of longer term outcomes i.e. a) do patients who access self managed therapies improve in the short term but relapse in the longer term, or b) do patients who access self-managed therapies maintain longer term outcomes and have less need for conventional CBT than those who remain on a waiting list prior to conventional CBT. These results will provide critical information concerning the longer term role of self-managed therapy packages for OCD compared to usual care with conventional CBT.

Potential participants (identified through screening waiting lists by a researcher at each of our sites, or through direct referral from professionals or direct advertisement to patients) will be invited to a telephone eligibility screen. If a participant meets the eligibility screen the researcher will give further details of the trial, send information leaflets and a consent form by post and offer them a face to face appointment (either in the clinical site or the patients own home) within 7 days from the telephone screen. At the face to face interview consent and baseline measures will be taken. Follow up interviews at 3, 6 and 12 months may be carried out over the telephone or questionnaires completed and returned by post. This will be dependent on participant preference should they be unable to meet face-to-face.

To ensure the removal of selection bias allocation will be concealed from the researchers completing assessments by randomisation through a central randomisation service. Details of eligible consenting patients will be entered onto a secure web based system administered by the York Clinical Trials Unit, which will then provide the treatment allocation. The system will generate an automatic email to both the site lead and the mental health worker of the participant’s treatment allocation. We have used this method successfully in one completed trial [13] and an ongoing mental health RCT [REEACT: the Randomised Evaluation of the Effectiveness and Acceptability of Computerised Therapy; NIHR HTA, PI Gilbody]. Allocation will involve minimisation on four important factors: OCD severity (moderate/severe), anti-depressant medication (yes/no), depression (mild/moderate/severe) and chronicity (duration of OCD – 0—5 years/ 6-10 years/ 10 years and over), with the aim of ensuring a balance across treatment arms.

To reduce detection bias, we will aim to blind researchers undertaking outcome assessments to participants’ treatment allocation. To facilitate blinding, we will use the following procedures: 1) ensuring that the outcome assessments conducted by research workers are completed on different days or locations from the clinical areas in which treatment is being conducted; and 2) asking participants to refrain from revealing their treatment allocation at follow-up assessments. Finally we will test blinding by asking research workers to guess the treatment allocation of the patient at each follow-up and to record the number of patients who inadvertently reveal their treatment allocation.

***Concurrent Process Evaluation:*** Criticism has been aimed at RCTs which only focus on pre-specified health outcomes [14]. Process evaluation within trials is recommended to examine key issues such as implementation, acceptability and feasibility which add to the understanding of the RCT results. This study will conduct a process evaluation to explore the barriers and facilitators of implementation by examining the extent to which: i) patients comply with treatment and ii) patients and health professionals find treatment acceptable.

***i) The extent to which patients comply with treatment***: A key aspect of any treatment is the extent to which users complete their agreed course of treatment including CBT between-session tasks. Treatment compliance will be examined through recording the number of sessions attended, and use of self-help materials. With cCBT we will collect automated recordings of the frequency and duration of cCBT use, and patient self reports of time spent between sessions doing CBT based tasks. In the bibliotherapy arm we will collect number, duration and mode of contact (telephone or face to face) via mental health worker records as well as patient diaries of between session work.

***i) The extent to which patients and health professionals find interventions acceptable:*** Successful implementation of research into NHS practise requires that new interventions are accepted and welcomed by both patients and mental health professionals. The 8-item Client Satisfaction Questionnaire (CSQ) will be administered to all patients at 3 and 6 months to assess acceptability. Qualitative interviews will be conducted at post-intervention with a subgroup of 10% of patients stratified by baseline severity in both active intervention arms of the trial across clinical sites. Patients will be sampled on characteristics including gender, ethnicity & outcome. We expect to conduct approximately 40-48 interviews (11-12 interviews at each site). We will also conduct exit interviews with participants who leave treatment early. A sample of 15-20 health professionals will be interviewed with the aim of exploring potential barriers and facilitators to implementing GSH and cCBT into clinical practice. Participants will be identified from health professionals who are working in our sites, and will include those who are delivering treatments in the trial, and those who work in our sites but are not treating patients as part of our trial, to maximise variation. The CSQ will be used to compare patient satisfaction across the treatment arms and, with other quantitative data, to identify predictors of satisfaction. Interviews will be conducted face-to-face, by telephone or by Skype dependent on participant preference and feasibility. Acceptability and exit interviews will be transcribed verbatim and data will be analysed using a framework analysis. An initial coding framework will be developed and transcripts checked against the framework to ensure that there are no significant omissions. Codes in each interview will be examined across individual transcripts as well as across the entire data set and allocated to the framework. Using aspects of the constant comparative method of analysis broader categories using linking codes will be developed across interviews. Data will be interpreted and analysed within the framework to structure patients’ views about each intervention and reasons for leaving the intervention early.

**3.4 Planned Interventions:**

*i) Experimental Group 1*: OCfighter ([www.ccbt.co.uk](http://www.ccbt.co.uk/)) is a commercially produced cCBT programme for people with OCD. OCfighter consists of a 9 step CBT approach (focussed on exposure and response prevention) to help people with OCD to design, carry out and monitor their treatment and progress. Participants randomised to OCfighter will be given an access ID and password to log into the system and will be advised to use the programme at least 6 times over a 12 week period. cCBT will be offered in one of three locations according to patient choice and local availability: (1) the patients’ own homes or that of friend and family (if they already have a computer and a broadband connection); (2) the CBT department in the clinical site where the patient is on the waiting list (if a computer in a private room operating on a weekly booking system can be provided). In this study we will actively encourage participants to use cCBT in their own home as the first option. This will maximise patient access and flexibility whilst respecting the importance of patient choice. Our experience in our ongoing cCBT trial in depression has already shown that his is by far the most preferred means of access. We will record the location of where trial participants randomised to cCBT access the internet.

Participants will also receive six, 10 minute brief scheduled telephone calls, via phone or face-to-face (depending on patient preference) from a mental health professional (total direct clinical input 60 minutes). The support offered will consist of a brief risk assessment, ensuring that they are able to and have accessed OC-Fighter, review progress and problem solve any difficulties which are impeding progress. Mental health professionals offering support to participants will have mental health training and are therefore trained in risk assessment and deliver low intensity interventions in primary care mental health but do not have specialist CBT skills. In a current HTA study of cCBT for depression (applicant Gilbody) we have written a detailed guide for mental health workers offering support to patients and we will adapt this for the mental health workers in this trial.

*ii) Experimental Group 2:* Bibliotherapy (Guided Self Help) will consist of a self help book ‘Overcoming OCD’: a workbook’ written by applicant Lovell [7]. Participants will receive weekly guidance from a mental health professional for 1 initial session of 60 minutes (either face to face or telephone dependent on patient preference) followed by up to 10 brief (30 minute) scheduled telephone, face to face (dependent on patient preference) sessions over a 12 week period (total direct clinical input 6 hours). The role of the mental health worker will be to conduct a semi-structured interview, devise patient centred goals and explain the structure and content of the book. They will support them to use the CBT interventions described in the book, review progress, pre-empt difficulties as they arise and engage in collaborative problem solving as required. The mental health professionals supporting bibliotherapy (guided self help) will be individuals who have had mental health training but do not have specialist CBT skills.

*iii) Comparator group 3:* Our control group will be a waiting list for conventional therapist-led CBT (in both primary and secondary care settings) as specified by the commissioning brief.

**3.5 Training:**

Training will be provided for the bibliotherapy (Guided Self Help) intervention by applicants (KL, SR, LG). These applicants have significant experience and expertise in conducting CBT, GSH and cCBT training to a range of mental health professionals in both trial and clinical practice settings locally, nationally and internationally. Training will be provided for OCfighter by applicants (KL, LG) in conjunction with STSolutions (manufacturers of OCfighter). Training will consist of 2 days for Guided Self Help and 2 days for cCBT and will be delivered at the clinical sites. The rationale for offering 2 days of training are so that mental health workers can familiarise themselves and work through both the cCBT package and the GSH workbook. Training will utilise a range of methods including small and large group work and skill practice with specific feedback using fictitious but typical cases of moderate and severe OCD. Training manuals will be written by the trial team and provided for both treatment arms.

*3.5.1 Supervision:* Supervision will be provided on a 2 weekly basis to the mental health professionals delivering the interventions by the trial team. Applicants KL (Manchester), SG, DM (York) MB (Sheffield), SR, LG (Norwich) are all experienced and/or accredited CBT therapists. Supervision will be face to face or telephone according to clinician preference.

**3.6 Planned inclusion/exclusion criteria:**

Our target population will be adults of 18 and above meeting DSM IV criteria for obsessive compulsive disorder (assessed using 6 OCD questions from the Mini-International Neuropsychiatric Interview (M.I.N.I.)), scoring 16 or over on the Yale Brown Obsessive Compulsive Checklist (YBOCS), and on a waiting list for therapist led CBT in either primary or secondary mental health care settings. In addition, patients must be able to read English at a level of 11 years and above. Our rationale for an inclusion threshold of 16 on the YBOCS is that this indicates a moderate level of OCD and is the cut off score used in most clinical trials. Clinical experience and previous studies which have included a consort diagram or detailed exclusion criteria suggest that only a minority of people are referred for treatment or excluded from trials with a YBOC less than 16 (e.g. 2.3% [1] 0% [15], 14% [16]). We will exclude patients, who are actively suicidal, have organic brain disease, are experiencing psychosis, who have a diagnosis (DSM IV) criteria of drug or alcohol misuse, are currently receiving a psychological treatment for OCD, or have literacy or language difficulties to an extent which would preclude them from reading written or web based materials or conversing with a health professional.

**3.7 Ethical arrangements**

This is a trial of human subjects receiving CBT interventions in innovative delivery formats. Research governance principles and ethical committee approvals bind all applicants and their institutions. We will ensure we adopt the highest standards of research conduct in this trial including involvement of user representation on the trial management group.

***Risks and anticipated benefits for trial participants and society:*** As with any comparative treatment trial, there is the potential for the new treatments (cCBT and bibliotherapy (Guided Self Help) to be less effective than established treatments. However patients will be on a waiting list for therapist delivered CBT and the proposed study will not restrict access to CBT when they reach the top of the waiting list. Although face to face CBT is recommended by NICE, the cost and complexity of this treatment means that access is problematic because insufficient treatment resources are available to meet demand. The OCTET trial will test the effectiveness and acceptability of new treatments for people with OCD who are on a waiting list for CBT and thus has the potential to improve access to care for the UK population in the future. People who become at risk of suicide during the intervention will be identified and directed/referred to appropriate care pathways. All health professionals delivering interventions in the study will have already received risk assessment training and we will supplement this by trial specific risk protocol training.

To ensure that randomised patients have the opportunity to complete the 12-week trial intervention prior to being offered a CBT appointment from their service we will ask them to wait. This may mean that they may have to wait slightly longer for their scheduled CBT appointment if the waiting time in their service is less than 12 weeks. All participants will have the opportunity to receive scheduled CBT following 12 weeks of treatment or waiting if randomised to the waiting list group.

***Informing potential trial participants of possible benefits and known risks:*** Patient information sheets will be written according to current guidelines provided by the NHS Integrated Research Application System (IRAS) and will be approved by the relevant ethics committee prior to the study commencing. Patient information leaflets will provide potential participants with information about the study, including potential benefits and risks of taking part in the trial. A further discussion with the researcher about the risks and benefits of the study will be provided prior to consenting to the trial.

***Obtaining informed consent from participants:*** Waiting lists will be screened by a researcher, clinical studies officer (CSO) or clinical/admin site contact and potential participants will be sent an information pack about the study. The pack will contain an information leaflet, a tear off ‘consent to contact form’, researcher details, and an SAE. The information leaflets and consent to contact form will adhere to and will have been agreed by the relevant IRAS ethics committee. Patients may also be identified through direct referral from professionals, direct advertisement to patients or by self-referral through local public advertising (local newspapers, local interest magazines, Trust websites, posters in local GP surgeries and community centres, social media site (e.g. facebook) presenting information about the trial at local OCD self-help groups and on local radio) or via a mailshot letter**,** sent to all patients currently on the waiting list. This letter will be sent with a copy of the patient information sheet (main trial) and consent to contact form. Participants who contact a researcher after receiving their contact details from an advertisement will then be sent a copy of the patient information sheet (main trial) and consent to contact form. Potential participants who consent to contact will be contacted by the site specific RA who will assess them for eligibility, discuss the trial and invite and answer questions. Written informed consent and baseline measures will be obtained prior to randomisation.

The OCTET research team based at the University of Manchester are keen to develop a research register containing OCTET participant contact details to give people the opportunity to take part in future research studies and be informed of OCD research findings. All participants who expressed an interest in the trial (and did not subsequently withdraw from follow-up) will be informed of the database by post and/or email. They will be given the opportunity to have their name included in the research register. Only individuals who return a completed opt-in form will be added to the register, their details will be help for 3 years.

***Management of relevant trial documentation:*** All data will be stored securely in line with local data management arrangements and accessed by the Trial Statistician. All paper records will be stored in secure storage facilities at the University of Manchester. Personal identifiable paper records will be stored separate from anonymised paper records. All electronic records will be stored on a password protected server within York Trials Unit and the University of Manchester. All contact information will be destroyed securely and immediately at the end of the trial.

***Proposed action to comply with EU directive and ‘The medicine for Human Use (Clinical Trials) regulations 2004:*** The study will be fully compliant with the EU directive and we will contact the relevant regulatory bodies in order to execute our study.

**3.8 Proposed sample size:**

Three pair-wise comparisons are planned between (i) self-managed therapy packages (cCBT v bibliotherapy), (ii) CBT waiting list and cCBT (iii) CBT waiting list and bibliotherapy.

In trials of non-pharmacological interventions it is recommended that variation between care-providers is considered in sample size estimation and statistical analysis. In this trial there could be variation by therapist (mental health workers) in the two self-managed therapies. For the comparisons of CBT waiting list with cCBT or bibliotherapy we have assumed between therapist variation in the active treatment corresponding to a partially-nested design [17]. For the comparison of cCBT v bibliotherapy the same set of therapists will "supervise" both interventions so the trial is therefore a cross-design [17]. Sample size is estimated using the methods described by Walwyn & Roberts [17].

We are not aware of directly relevant estimates for intra-cluster correlation coefficient (ICC) for therapist required for sample size for comparison (ii) & (iii) or the ICC between treatments within therapist for comparison (i). We expect the ICC between treatments within therapist to be less than half the ICC between therapists. With a total sample size of 432 clients and 24 therapist (average of 6 therapists per clinical site) each with a caseload of 6 cCBT and 6 bibliotherapy clients the trial will have a power greater than 80% to detect a difference of 3 YBOC points for each comparison provided the ICC for therapist does not exceed 0.06 and the ICC for treatment within therapist does not exceed 0.015. This calculation assumes a 85% follow-up rate to 6 months (87% was achieved within Lovell et al [1]), a 1.67% significance level to maintain a 5% sig level for three pair-wise comparisons, SD for the primary outcome YBOC at 6 months of 7.3 unit, a correlation between baseline YBOCS and 6 month YBOC of 0.43 [1]. In the event that the ICC for therapist is less than 0.1 and the ICC for treatment within therapist is less than 0.05 (which would represent unexpectedly large values), the power is still greater than 75%. By virtue of involving a comparatively large number of therapists the trial is therefore robust again larger ICC values for therapist.

**3.9 Recruitment strategy:**

Our trial will recruit a minimum of 432 patients with OCD across four clinical sites (assuming a 15% attrition rate, total N=368) with each site recruiting patients over a 27 month recruitment period (including the 9-month pilot phase). All sites will be recruiting from both primary and secondary care services (i.e. in some primary care services high intensity therapists offer full CBT and refer onto secondary care if patients fail to improve while in other services people with OCD are immediately referred to CBT delivered in secondary care). Referral rates for all the clinical sites for people with OCD range from between 60-100 patients per year. In a previous study of psychological treatment with OCD 76% of people referred for the study were randomised [1]. Each site has a designated site lead Manchester (Bee), Norwich (Reynolds), York (McMillan), and Sheffield (Barkham). To ensure recruitment we have checked waiting lists in both primary and secondary care in our clinical sites and waiting lists range from 4 to 18 months. Strong links are in place on all sites between clinical and academic staff and three of our four sites have an applicant currently treating people with OCD which will further enhance engagement with recruitment sites. In the lead site in Manchester we have had discussions with the MHRN regarding recruitment with this study if successful. The MHRN (NW hub) includes 6 NHS Trusts (including Manchester Mental Health and Social Care NHS Trust) serving a population of 3.45 million. We have agreed that if funded we will recruit from all 6 NHS trusts and to assist with recruitment in this large geographical area clinical support officers (CSOs) will be trained to conduct telephone eligibility interviews with potential participants. Of the 6 trusts in the NW hub we have agreement in principle from 4 of the NHS Trusts to host the research. We will also apply for adoption by the Mental Health Research Network and link with the Primary Care Research Network to assist with recruitment from PCTs in our clinical sites to ensure that we fulfil our recruitment target. We have a proven track record in recruiting to trials on time and within specified targets [1,18]. Our trial of cCBT uses the support network outlined above and is currently recruiting ahead of target. We have included support costs agreed and negotiated with services to compensate for any additional time involved in the process of recruitment. To meet our recruitment target we will need to recruit 17 patients per month across our 4 sites with a site recruitment target of 4-5 patients per month at each site.

**Attrition:** Attrition (or loss to follow up) occurs when a participants’ data cannot be collected for some reason and can lead to bias in RCTs [19,20]. Our estimate for attrition at the primary outcome point is 15%, based on the relevant literature of face to face OCD, cCBT and self help. In our previous trial of OCD [1] which compared 10 hours of telephone delivered CBT with face to face CBT we retained 94% of patients at post intervention and 90% at 6 month follow up. In a previous trial of guided self help by some of our team [21] we achieved follow up rates of 90% at 3 month follow up.

In keeping with the philosophy of an intention-to-treat analysis we will make every effort to follow people up regardless of whether they adhered to the treatment protocol. As those who do not adhere to the treatment protocol tend to be less adherent with outcome assessments, we will establish special procedures and make targeted efforts to achieve high rates of follow up and ensure we minimise differential attrition between treatment arms. We will explore the effect of any bias through a sensitivity analysis.

**3.10 Statistical analysis:**

A full statistical analysis plan for the analysis of primary and secondary outcome measures including any sub-group analyses will be prepared and presented to the TSC (Trial Steering Committee) prior to the commencement of the trial analysis. During the recruitment and follow-up period regular reports will be prepared for the TSC/DMEC on data quality.

Statistical analysis of outcome will follow intention-to-treat principles: patients will be analysed according to randomised group and outcome data will be sought and included in the analysis for all patients irrespective of completion of treatment. Preliminary statistical analysis will model the pattern of missing data in terms of baseline characteristics of patients.

Statistical analyses of the primary outcome measure, YBOC at 3 months, will be based on a mixed model analysis with random effects for therapist and covariates including baseline YBOC and variables potentially predictive of outcome including minimisation variables. This will be used to compare all three arms. Conditional on significance at a 5% level, pairwise comparisons between arms will be carried out using a Bonferroni corrected p-value. Similar analyses will be carried out for quantitative secondary outcomes. A logistic mixed model will be use to compare the percentage of patients requiring full CBT between treatment adjusted for baseline severity.

**3.11 Economic analysis:**

Little evidence exists regarding the service use or costs associated with OCD, or the cost-effectiveness of alternative treatment strategies [22-24], with the exception of a small number of cost of illness or costing studies [25-28], productivity loss [27] and caregiver burden [29] estimates. Clinical evaluations of treatment alternatives for OCD that have included a cost or cost-effectiveness component have been severely limited to the cost of the interventions under evaluation, with no data collected on the impact of the treatment options on the use of other health and social services, patient and family costs or productivity losses [11,30,31]

The proposed economic evaluation will take a societal perspective. Data will be collected on the use of all hospital and community health and social services, productivity losses and costs to patients/families. To the best of our knowledge, no OCD specific tool for measuring use of services and other resources exists. Instead, the Adult Service Use Schedule (AD-SUS), a generic measure developed by the applicants (SB) and successfully applied to a range of studies of adult mental health services [32-34] will be adapted to ensure it adequately captures resources appropriate to OCD. The AD-SUS will initially be adapted for OCD through review of relevant literature and discussions with the clinical team. The adapted version will then be tested at baseline interview with participants in the pilot phase to ensure all important resources are captured. Intervention resources will be collected from therapist records to ensure accuracy and avoid unblinding research assessors.

The unit cost of study interventions will be calculated directly using established methods of micro-costing [35]. Calculations will require data on therapist salaries, including appropriate overheads and employers’ oncosts (national insurance and superannuation), working time and estimates of the ratio of direct face-to-face to indirect time. The cost of cCBT will require information on the licensing costs, plus data on any additional purchases of equipment required. For all other health and social services, nationally applicable unit costs will be applied [36-38]. Productivity losses will be calculated using the human capital approach, which involves multiplying days off work due to illness by the individual’s salary [39]

Differences in mean costs will be analysed using standard parametric t-tests with the validity of results confirmed using bias-corrected, non-parametric bootstrapping (repeat re-sampling) [40]. Despite the skewed nature of cost data, this approach is recommended to enable inferences to be made about the arithmetic mean [41].Tests will be adjusted for baseline cost, baseline YBOC score and variables potentially predictive of outcome including minimisation variables.

Cost-effectiveness will be explored in terms of incremental cost per quality adjusted life years; calculated using the EQ-5D measure of health related quality of life, and using rules of dominance and extended dominance for a three-arm comparison [42]. The primary perspective of the analysis will be the NHS/Personal Social Services perspective preferred by the National Institute of Health and Clinical Excellence. Secondary analyses will take a societal perspective. In line with the key objectives of the proposed trial, the following cost-effectiveness analyses will be undertaken:

1. Self-managed CBT (cCBT and bibliotherapy) compared to no active intervention (i.e. CBT waiting list) in the management of OCD patients in the short-term (3 or 6 month follow up, dependent on pilot phase results, as described above).
2. Self managed therapies plus usual care (waiting list plus access to therapist led full CBT) versus usual care alone at 12 month follow up.

Nonparametric bootstrapping from the costs and effectiveness data will be used to generate a joint distribution of incremental mean costs and effects for the three arms. This will then be used to calculate the probability that each of the treatments is the optimal choice, subject to a range of possible maximum values (ceiling ratio) that a decision maker might be willing to pay for a unit improvement in outcome. Cost-effectiveness acceptability curves are presented by plotting these probabilities for a range of possible values of the ceiling ratio [43]. These curves are a recommended decision-making approach to dealing with the uncertainty that exists around the estimates of expected costs and expected effects associated with the interventions under investigation [44]

**3.12 Proposed outcome measures:**

The primary outcome will be OCD symptoms as measured by the Yale Brown Obsessive Compulsive Checklist YBOCS [12] observer-report version. Secondary outcomes will include self-reported health-related quality of life (SF-36), self-reported OCD symptoms (YBOCs self-rated), generic mental health (CORE-OM), depression (PHQ9), anxiety (GAD-7), functioning (WSA), health-related quality of life (EQ5D), employment status (IAPT Employment Status questions A13 –A15), patient satisfaction (CSQ) and acceptability (qualitative interviews), and % patients not improved or partially improved requiring more intensive CBT. Our primary outcome point (3 month follow up) will be determined by the pilot study. A 6 and 12 month follow up will also be included.

**4. Research Governance:**

*Trial sponsorship:* The University of Manchester has agreed to act as sponsor.

*Trial management:* The trial management group will meet quarterly and will include the principal investigator, all other investigators, and trial manager to discuss the progression and day to day management issues of the trial. The principal investigator will be responsible for the overall leadership, management and outputs of the study. The principal investigator will maintain a log of the key milestones to be achieved against the timetable (as outlined in the section below). Progress of these milestones and corresponding timetable will be reported at the quarterly meetings to ensure progression of the trial and to agree corrective action if necessary. The trial manager (based in Manchester) will be responsible for the day to day running and coordination of the study and will be accountable to the principal investigator. Their role will include obtaining ethics and research governance approval, coordinating the collection of data, preparation of meetings and assisting with the writing and execution of the procedures and policies for the trial, producing initial drafts for research papers and disseminating the study’s findings. They will also be responsible for ensuring recruitment is on target by collating monthly reports from each site researcher and reporting this to the PI, such a monitoring system will allow for corrective action to be taken at the earliest opportunity. Site research workers supervised by the site lead will be responsible for recruitment, the conduct of eligibility and follow up assessments and data collection. They will also be responsible for conducting qualitative acceptability interviews at post treatment. Data will be securely stored locally and entered on a secure electronic recording system. Data will be checked by the trial manager for errors and data completion prior to transfer to the trial statistician. The trial statistician will be responsible for cleaning the data, conducting the analysis and sending the data to the DMEC. We are using the services of the York Trials Unit who will provide a secure allocation of trial participants to treatment and will be responsible for the minimisation process. They will also provide a comprehensive data management service including devising the electronically secure recording system.

*Trial Steering Committee:* A trial steering committee (TSC) will be established and comprise of an independent chair who has expertise in both trials and OCD and two other independent members including a user representative who has had lived experience with OCD and a clinician working with people with OCD.

*Data Monitoring and Ethics Committee (DMEC):* A DMEC will be established to assess the progress and safety of the study. It will consist of members external to the study team including a statistician, a clinician and an expert in health services trials. A DMEC report template will be devised for reporting purposes and agreed by the DMEC committee prior to the commencement of the study.

**5. Project timetable and milestones:**

| **Date** | **Milestones** |
| --- | --- |
| Months 1-3 | Apply for ethics and governance approval for all sites  Apply for adoption by MHRN  Advertise and appoint trial manager and Research Assistants at each site  Develop procedures and policies for the conduct of the trial  Conduct training in GSH and cCBT and develop training manuals  Initial adaptation of AD-SUS  Appoint members to the trial steering committee and DMEC |
| Months 4-13 | Recruitment to pilot study (n=153; Estimated recruitment rate=17/mth)  Pilot testing of AD-SUS  Delivery of interventions  Report to DMEC, TSC and interim report to HTA regarding continued funding. |
| Months 14 -43 | Proceed to full trial after agreement from HTA  Recruitment and intervention (months 14-31; (n 279; Estimated recruitment rate=17/mth)  Data collection at 3, 6 and 12 month follow-up (Final follow up month 43)  Conduct and analyse acceptability interviews |
| Months 43-48 | Closing of database  Data cleaning, statistical & economic analysis  Prepare final report for HTA  Send trial participants details of study findings  International conference to present main findings  Final report disseminated as a publication, user websites and a conference for dissemination of final results. |

**6. Expertise:**

Our multidisciplinary research team has the necessary expertise and are qualified to deliver the proposed research. Together the research team has a broad range of methodological expertise in trials, statistics, health economics, qualitative interviewing. We have conducted pilot work and RCTs of adults and young people with OCD investigating traditionally delivered CBT as well as more accessible technologies for CBT delivery such as the telephone, cCBT and guided self-help manuals (Reynolds, Lovell, Gega, Roberts, Byford, Hardy). We have extensive clinical expertise in traditionally delivered CBT as well as CBT based remote technologies with OCD (Lovell, Gega). We have expertise in developing (Lovell,) and evaluating (Lovell, Bee) structured self-help materials for OCD, depression and agoraphobia. Site leads will provide supervision and personal development for RA’s at each site.

- *Professor Karina Lovell (PI)* is professor of mental health, an experienced health care researcher, cognitive behaviour therapist and member of the NICE OCD guideline committee group. She has led on and contributed to previous clinical trials of psychological intervention studies. *Contribution:* overall management of the trial, development and delivery of training and supervision.
- *Professor Michael Barkham* has carried out numerous quantitative and qualitative studies of psychotherapy processes and outcomes over the past 20 years with a particular focus on the therapeutic alliance. He has completed the Newcastle CBT postgraduate course and is currently Joint Editor of the British Journal of Clinical Psychology and directs the Psychological Services Research Centre at the University of Sheffield. *Contribution:* site lead
- *Dr Peter Bower* is an experienced health services researcher working at the National Primary Care Research and Development Centre and the NIHR School of Primary care Research. He has expertise in the design of randomised trials of psychological therapy in primary care, recruitment and retention issues in trials, and the evaluation of complex interventions, including stepped care and self managed therapies for mental health problems. *Contribution:* methodological, design and analysis, policies and procedures for the trial.
- *Dr Sarah Byford* is a health economist with particular expertise in the economic evaluation of mental health services. She has led a large number of economic evaluations in related areas, including evaluation of CBT and mindfulness based cognitive therapy for depression and individual and group-based therapies for deliberate self harm. *Contribution:* Health economics.
- *Dr Penny Bee* is an experienced health services researcher and has examined innovative ways of delivering mental health services. She has expertise in the evaluation of complex interventions including self-help packages for mental health problems and remotely-communicated psychotherapies. *Contribution:* site lead.
- *Dr Lina Gega* is a lecturer in mental health and a practising CBT specialist in primary care has had extensive experience in computer-aided self-help and in CBT delivery, supervision and training. *Contribution:* development and delivery of training and supervision.
- *Professor Simon Gilbody* is an experienced health service researcher and honorary consultant clinical psychiatrist and cognitive behaviour therapist. He has expertise in evidence synthesis and trial design. He is currently Editor of the Cochrane Depression Group. *Contribution:* development and delivery of training and supervision.
- *Professor Gillian Hardy* is an experienced psychotherapy process researcher. She has extensive experience of working with psychotherapy trials and was Director of a Research CBT Clinic in Leeds. *Contribution:* process evaluation and qualitative interviews and analysis.
- *Ms Nicky Lidbetter* is the Chief Executive of two user-led mental health charities; Anxiety UK (formerly National Phobics Society) and Self Help Services (a 3^rd^ sector organisation) which provides cCBT to a number of NHS Trusts in Greater Manchester. She provided input into the first NICE technology appraisal for cCBT and has expertise in primary care mental health services by third sector organisation - particularly around the delivery of IAPT services by third sector organisations. She is trained to post graduate level in brief therapeutic interventions. Lidbetter has personal experience of anxiety and through Self Help Services she provides supported cCBT services to many PCTs in the Greater Manchester area. *Contribution:* service user input and development and delivery of training*.*
- *Professor Shirley Reynolds* is an experienced researcher in psychotherapy research and has specific expertise in both clinical and research issues with OCD. *Contribution:* site lead and development and delivery of training and supervision.
- *Dr Christopher Roberts* is a statistician and health sciences researcher. He has significant expertise in trial design and analysis. *Contribution:* design and statistical input
- *Dr Dean McMillan* is an experienced Clinical Psychologist, with further specialist training in Cognitive Behaviour Therapy (Newcastle Postgraduate Diploma in CBT).*Contribution:* site lead and development of training and supervision
- *Dr Pat Mottram* is Research and Effectiveness Manager at Cheshire and Wirral Partnership NHS Trust. *Contribution:* Interfacing between the trial and the clinical site and ensuring recruitment to the trial is has a high priority *.*
- Collaborators: Professor David Torgerson is a leading health services researcher and Director of the York Trial Unit. Via this role he has specific experience in the design, conduct and analysis of primary care led trials.

**7. Service users:**

Service user input, involvement and dissemination of the proposed study comes from Nicky Lidbetter (applicant) Chief Executive of Anxiety UK. We have strong links with Anxiety UK and currently have a number of funded (including both a NIHR programme grant and an HTA grant) and unfunded collaborative studies (a qualitative study of CBT telephone acceptability for anxiety and depression, applicants Lovell, Bee & Lidbetter). In addition we will also invite members of a CCBT OCD sub group of the Patient and Public Involvement in Research Group in Norfolk who have agreed to be part of our steering committee. Our service user representation will also include assisting us in developing the interview schedules for the acceptability interviews. We will also offer service users training in interviewing if they wish to conduct a proportion of the interviews and they will contribute to the analysis of the interviews and assist in the writing of publications. We are strongly committed to user involvement in our research endeavours and applicants (Lovell, Bower) have conducted a workshop under the auspices of the Institute of Health Sciences of engaging meaningful user involvement in research. A proportion of Ms Lidbetter’s time will be funded through the grant. A consultancy fee will be paid to participating members of the patient and public involvement in research group.

**8. Justification of support required:**

Each of the four sites will require a research worker (grade 6) WTE for the first 3 years of the trial and reducing to 0.4 for the final year to facilitate recruitment, conduct eligibility and follow up assessments. Site researchers will also be responsible for conducting qualitative acceptability interviews at post treatment. Byford will lead the economic analysis supported by a research worker for 0.25 WTE. A 0.8 WTE trial manager will be based at the University of Manchester and will be responsible for the day to day running and coordination of the trial. A 0.1 WTE trial manager will be based in the York Clinical Trials Unit to co ordinate and supervise data management and to interface with the rest of the trial sites. In addition to staff costs above we will need funding to purchase sufficient internet delivered OCD treatment packages (OCfighter) for all the people taking part in the study, costs for York clinical trials unit for randomisation, data entry, and cleaning and preparation of data for analysis. We will also need funds for travel expenses of the trial team and assessment visits, and costs associated with project administration (e.g. postage and office supplies). NHS sites in each of the geographical areas taking part in our study will need money towards their administrative costs, training time, supervision, utilities, and workspace and overhead charges during the time that we are working in or at their buildings.

**9. References:**

1. Lovell K, Cox D, Haddock G, Jones C, Raines D, Garvey R. Telephone administered cognitive behaviour therapy for treatment of obsessive compulsive disorder: Randomised controlled non-inferiority trial. British Medical Journal 333:883,2006
2. Office for National Statistics. Statistical Bulletin:: Internet Access [http://www.statistics.gov.uk/pdfdir/iahi0809.pdf; Last accessed 17^th^ March 2010]
3. Murray C. J. L, Lopez A. D. The global burden of disease. Cambridge, MA: The Havard School of Public Health on behalf of the World Health Organization and The World Bank; 1996.
4. Bebbington P. E. Epidemiology of obsessive-compulsive disorder. British Journal of Psychiatry 35(Suppl.):2–6. 1998.
5. Torres A. R, Prince M. J, Bebbington P. E, Bhugra D, Brugha T. S, Farrell M. Obsessive-compulsive disorder: Prevalence, comorbidity, impact, and help-seeking in the British National Psychiatric Morbidity Survey of 2000. American Journal of Psychiatry 163(11):1978–1985. 2006.
6. National Institute for Clinical Excellence. Obsessive-compulsive disorder: Core interventions in the treatment of obsessive-compulsive disorder and body dysmorphic disorder. London: HMSO; 2005
7. Lovell K, Ekers D, Fullford J, Baguely C, Bradshaw T. A pilot study of a self-help manual with minimal therapist contact in the treatment of obsessive-compulsive disorder. Clinical Effectiveness in Nursing 2005, 8(2):122–127.
8. Fritzler B. K, Hecker J. E, Loose M. C. Self-directed treatment with minimal therapist contact: Preliminary findings for obsessive-compulsive disorder. Behaviour Research and Therapy 1997, 35:627–631
9. Taylor S, Thordarson DS, Spring T, Yeh A, Corcoran K, Eugster K, and Tisshaw C (2003). Telephone-Administered Cognitive Behavior Therapy for Obsessive-Compulsive Disorder. Cognitive Behaviour Therapy Vol 32, No 1, pp. 13–25, 2003
10. Kaltenthaler E, Brazier J, De Nigris E, Tumur I, Ferriter M, Beverley C, et al. Computerised cognitive behaviour therapy for depression and anxiety update: a systematic review and economic evaluation. Health Technol Assess 2006;10(33).
11. McCrone P, Marks IM, Greist JH, Baer L, Kobak KA, Wenzel KW, Hirsch MJ (2007). Cost-effectiveness of computer-aided behaviour therapy for OCD. Psychother Psychosom, 76:249-250
12. Goodman, W.K., Price, L.H., Rasmussen, S.A. et al. The Yale-Brown Obsessive Compulsive Scale. Arch Gen Psychiatry 1989, 46:1006-1011.
13. Richards D, Lovell K, Gilbody S, Gask L, Torgeson D, Barkham M, Bland M, Bower P, Lankshear A, Simpson A, Fletcher J, Escott D, Hennessy S, Richardson R. (2007) Collaborative Care for Depression in UK Primary Care: a randomised controlled trial. Psychological Medicine, 2007 Doi: 10.1017/s0033291707001365
14. Daniel J. Martin, John P. Garske, M. & Davis K. Relation of the Therapeutic Alliance with Outcome and Other Variables: A Meta-Analytic Review. Journal of Consulting and Clinical Psychology 2000, 68:438-450.
15. Anderson, Rebecca A; Rees, Clare S. Group versus individual cognitive-behavioural treatment for OCD: A controlled trial. Behaviour Research and Therapy 200, 45(1): 123-137
16. Tolin KL, Hannan DF, Maltby S, Diefenbach N, Worhunsky P, Brady RE. A RCT of self-directed versus therapist-directed CBT for OCD patients with prior medication trials. Beh Ther, 2007, 38 (2):179-191.
17. Walwyn R, Roberts C. Therapist variation within randomised trials of psychotherapy: implications for precision, internal and external validity. Stat Methods Med Res. 2009, 00: 1–25
18. Chew-Graham CA, Lovell Karina, Roberts C, Baldwin B, Morley M, Burns A, Burroughs H. Achieving target recruitment in a primary care trial: lessons from PRIDE. Primary Health Care Research & Development, 2007 8: 264-270.
19. Dumville JC, Torgerson DJ, Hewitt, CE. Reporting attrition in randomised controlled trials. BMJ 2006; 332:969-971
20. Heneghan C, Perera1 R, Ward A, Fitzmaurice D, Meats E and Glasziou P. Assessing differential attrition in clinical trials: self-monitoring of oral anticoagulation and type II diabetes BMC Medical Research Methodology 2007, 7:18doi:10.1186/1471-2288-7-18
21. Mead, N; MCDonald, W; Bower P; Lovell K; Richards D, Roberrts C, Bucknall A (2005). The clinical effectiveness of guided self-help versus waiting-list control in the management of anxiety and depression: a randomized controlled trial. Psychological Medicine, **35** , pp 1633-1643 doi:10.1017/S003329170500560X
22. Bower, P., Garralda, E., Kramer, T., Harrington, R., & Sibbald, B. (2001). The treatment of child and adolescent mental health problems in primary care: a systematic review. Family Practice, vol. 18, no. 4, pp. 373-382.
23. Knapp, M., Henderson, J., & Patel, A. (2002). Costs of obsessive-compulsive disorder: a review, in Obsessive-compulsive disorder, M. Maj et al., eds., John Wiley & Sons Ltd, Chichester, pp. 253-275.
24. Barlow, J. H., Ellard, D. R., Hainsworth, J. M., Jones, F. R., & Fisher, A. (2005). A review of self-management interventions for panic disorders, phobias and obsessive-compulsive disorders. Acta Psychiatrica Scandinavica, vol. 111, pp. 272-285.
25. Dupont R. L, Rice D. P, Shiraki S, Rowland C. R. Economic costs of obsessive compulsive disorder. Medical Interface 8:102–109. 1995
26. Leon, A. C., Portera, L., & Weissman, M. M. (1995). The social costs of anxiety disorders. British Journal of Psychiatry, vol. 166, no. Suppl. 27, pp. 19-22.
27. Hollander, E., Broatch, J., Himelein, C., Rowland, C., Stein, D., & Kwon, J. (1997). Psychosocial function and economic costs of obsessive-compulsive disorder. CNS Spectrums: The International Journal of Neuropsychiatric Medicine, vol. 2, no. 10, pp. 16-25.
28. National Institute of Mental Health (2000). A real illness: obsessive-compulsive disorder, National Institute of Mental Health, National Institutes of Health, US Department of Health and Human Services, Bethesda MD, NIH Publication No. 00-4676.
29. Kalra, H., Kamath, P., Trivedi, J. K., & Janca, A. (2008). Caregiver burden in anxiety disorders. Current Opinion in Psychiatry, vol. 21, pp. 70-73.
30. Marks, I. M., Mataix-Cols, D., Kenwright, M., Cameron, R., Hirsch, S., & Gega, L. (2003). Pragmatic evaluation of computer-aided self-help for anxiety and depression. British Journal of Psychiatry, vol. 183, pp. 57-65.
31. Gega, L., Marks, I., & Mataix-Cols, D. (2004). Computer-aided CBT self-help for anxiety and depressive disorders: experience of a London clinic and future directions. Journal of Clinical Psychology, vol. 60, pp. 147-157.
32. Barrett, Byford, Crawford, Patton, Drummond, Henry, Touquet. Cost-effectiveness of referral to an alcohol health worker in patients attending an accident and emergency department: a decision-making approach. Drug and Alcohol Dependence, 2006, 81:47-54.
33. Bower, Byford, Sibbald et al. Randomised controlled trial of non-directive counselling, cognitive-behaviour therapy, and usual general practitioner care for patients with depresion. II: Cost effectiveness. Br Med J 2000, 321 (7273):1389-92.
34. Byford, Knapp, Greenshields,Ukoumunne, Jones, Thompson, Tyrer, Schmidt, Davidson, Catalan. Cost-effectiveness of brief cognitive behaviour therapy versus treatment as usual in recurrent deliberate self-harm: a rational decision making approach. Psychological Medicine 2003,33: 977-986.
35. Drummond, M. F., O'Brien, B., Stoddart, G. L., & Torrance, G. W. (1997). Methods for the economic evaluation of health care programmes, Second edn, Oxford University Press, Oxford.
36. Curtis, L. (2008). The unit costs of health and social care 2008 Personal Social Services Research Unit, University of Kent at Canterbury.
37. Department of Health (2008). NHS reference costs 2008 Department of Health, London.
38. British Medical Association & Royal Pharmaceutical Society (2008). British National Formulary British Medical Association, London.
39. Koopmanschap, M. A. & Rutten, F. (1996). A practical guide for calculating indirect costs of disease. Pharmacoeconomics, vol. 10, pp. 460-466.
40. Efron, B. & Tibshirani, R. J. (1993). An introduction to the bootstrap Chapman & Hall, New York.
41. Thompson, S. G. & Barber, J. A. (2000). How should cost data in pragmatic randomised trials be analysed? British Medical Journal, vol. 320, pp. 1197-1200.
42. Johannesson,M. & Weinstein,M.C. (1993) On the decision rules of cost-effectiveness analysis. Journal of Health Economics, vol. 12, pp. 459-467.
43. Fenwick, E., Sculpher, M., & Claxton, K. (2000). Representing uncertainty: the role of cost-effectiveness acceptability curves, Centre for Health Economics, York.
44. Claxton, K. (1999). The irrelevance of inference: a decision-making approach to the stochastic evaluation of health care technologies. Journal of Health Economics, vol. 18, pp. 341-364.
